# Reporting Cultural Adaptation in Psychological Trials – The RECAPT criteria

**DOI:** 10.32872/cpe.6351

**Published:** 2021-11-23

**Authors:** Eva Heim, Ricarda Mewes, Jinane Abi Ramia, Heide Glaesmer, Brian Hall, Melissa Harper Shehadeh, Burçin Ünlü, Schahryar Kananian, Brandon A. Kohrt, Franziska Lechner-Meichsner, Annett Lotzin, Marie Rose Moro, Rahmeth Radjack, Alicia Salamanca-Sanabria, Daisy R. Singla, Annabelle Starck, Gesine Sturm, Wietse Tol, Cornelia Weise, Christine Knaevelsrud

**Affiliations:** 1 Institute of Psychology, University of Lausanne, Lausanne, Switzerland; 2 Department of Psychology, University of Zürich, Zürich, Switzerland; 3 Outpatient Unit for Research, Teaching and Practice, Faculty of Psychology, University of Vienna, Vienna, Austria; 4 National Mental Health Programme – Ministry of Public Health, Beirut, Lebanon; 5 Department of Clinical, Neuro- and Developmental Psychology, Amsterdam Public Health Research Institute, Vrije Universiteit Amsterdam, Amsterdam, The Netherlands; 6 Department of Medical Psychology and Medical Sociology, University of Leipzig, Leipzig, Germany; 7 Global Public Health, New York University Shanghai, Shanghai, China; 8 PsyQ, Parnassia Psychiatric Institute, The Hague, The Netherlands; 9 Department of Clinical Psychology and Psychotherapy, Goethe University Frankfurt, Frankfurt, Germany; 10 Department of Psychiatry and Behavioral Sciences, George Washington University, Washington, DC, USA; 11 Department of Psychiatry and Psychotherapy, University Medical Center Hamburg-Eppendorf, Hamburg, Germany; 12 Inserm, Hôpital Cochin, AP-HP, Paris University, Paris, France; 13 Future Health Technologies, Singapore-ETH Centre, Campus for Research Excellence and Technological Enterprise (CREATE), Singapore, Singapore; 14 Campbell Family Mental Health Research Institute, Toronto, Canada; 15 Department of Psychiatry, Temerty Faculty of Medicine, University of Toronto, Toronto, Canada; 16 Lunenfeld Tanenbaum Research Institute, Toronto, Canada; 17 Laboratoire Cliniques Psychopathologique et Interculturelle LCPI EA 4591, Université Toulouse II - Jean Jaurès, Toulouse, France; 18 Section for Global Health, Department of Public Health, University of Copenhagen, Copenhagen, Denmark; 19 Division of Clinical Psychology and Psychotherapy, Department of Psychology, Philipps-University of Marburg, Marburg, Germany; 20 Department of Education and Psychology, Freie Universität Berlin, Berlin, Germany; Philipps-University of Marburg, Marburg, Germany

**Keywords:** cultural adaptation, reporting criteria, randomised controlled trials, common mental disorders, psychological interventions

## Abstract

**Background:**

There is a lack of empirical evidence on the level of cultural adaptation required for psychological interventions developed in Western, Educated, Industrialized, Rich, and Democratic (WEIRD) societies to be effective for the treatment of common mental disorders among culturally and ethnically diverse groups. This lack of evidence is partly due to insufficient documentation of cultural adaptation in psychological trials. Standardised documentation is needed in order to enhance empirical and meta-analytic evidence.

**Process:**

A “Task force for cultural adaptation of mental health interventions for refugees” was established to harmonise and document the cultural adaptation process across several randomised controlled trials testing psychological interventions for mental health among refugee populations in Germany. Based on the collected experiences, a sub-group of the task force developed the reporting criteria presented in this paper. Thereafter, an online survey with international experts in cultural adaptation of psychological interventions was conducted, including two rounds of feedback.

**Results:**

The consolidation process resulted in eleven reporting criteria to guide and document the process of cultural adaptation of psychological interventions in clinical trials. A template for documenting this process is provided. The eleven criteria are structured along A) Set-up; B) Formative research methods; C) Intervention adaptation; D) Measuring outcomes and implementation.

**Conclusions:**

Reporting on cultural adaptation more consistently in future psychological trials will hopefully improve the quality of evidence and contribute to examining the effect of cultural adaptation on treatment efficacy, feasibility, and acceptability.

## Highlights

- Adaptation starts with defining the target population along cultural and socio-demographic criteria.
- Literature review and formative research are used to define target symptoms, syndromes, needs, and context.
- Using a standardized documentation system to structure the adaptation process is recommended.
- Documentation includes results of formative research and adaptation of treatment elements.

Psychotherapies developed in Western, Educated, Industrialized, Rich, and Democratic (WEIRD; Henrich et al., 2010) societies may not or only partly be relevant to cultural groups or ethnic minorities who differ from the former in terms of cultural values, norms, or illness concepts. Evidence indicates that cultural adaptation of psychological interventions for the treatment of common mental disorders increases their acceptability and efficacy (Benish et al., 2011; Chowdhary et al., 2014; Hall et al., 2016; Harper Shehadeh et al., 2016). There is a large variety of target populations, psychological interventions and settings where cultural adaptation is applied, from low-intensity interventions in humanitarian settings (Perera et al., 2020) to higher-intensity interventions through the internet (Knaevelsrud et al., 2015) or face-to-face (Hinton et al., 2012), to mention only a few. Most cultural adaptation studies use a top-down approach, in which existing psychological interventions developed for one cultural group are adapted for another one. Few studies use a bottom-up approach to develop new interventions based on culturally specific symptoms or syndromes (Hall et al., 2016; Hwang, 2006).

So far, there are no standard criteria for documenting bottom-up and top-down cultural adaptations in clinical trials testing psychological interventions (in short: psychological trials). A more detailed standard documentation is key to obtain more reliable information regarding the effect of cultural adaptation on treatment efficacy, feasibility, and acceptability. In this paper, we suggest a set of reporting criteria for this purpose. First, we outline the theoretical and empirical background. Thereafter, the reporting criteria are introduced. More detailed information on the background, the development of the reporting criteria, and the use of these criteria, can be found in Appendix A (see Supplementary Materials).

## Background

In the *Lancet Commission on Culture and Health*, culture is defined as follows: “Culture, then, can be thought of as a set of practices and behaviours defined by customs, habits, language, and geography that groups of individuals share” (Napier et al., 2014, p. 1609). In Appendix A (Supplementary Materials), we provide additional definitions of culture. These definitions highlight that culture refers to shared systems of understanding and engaging with the world, which extends beyond language and ethnicity to include political, economic, environmental, and other contexts that shape these patterns of shared experience.

For instance, this means that translation from English into Spanish is unlikely to be sufficient to address the needs of residents of Barcelona, Venezuelan refugees in Colombia, and first generation Salvadoreans immigrated to the United States. Conversely, because culture is strongly tied to context, many of the adaptations done for Syrian refugees in urban host communities in Jordan may be helpful for Venezuelan refugees in urban host communities in Colombia, despite the language of adaptation being entirely different. Cultural adaptation, therefore, refers to enabling an intervention to produce its desired psychological effect with a particular group in a specific context.

Several frameworks for cultural adaptation of evidence-based interventions exist (e.g., Applied Mental Health Research [AMHR] Group at Johns Hopkins University, 2013; González Castro et al., 2010; Perera et al., 2020), all of which have been developed mainly for clinical practice. These frameworks have in common that they use stage models which include assessment, selection of the intervention (components), adaptation, piloting, and implementation. Such stage models provide guidance on the process of cultural adaptation (i.e., *how* to adapt). With regard to content of cultural adaptation (i.e., *what* to adapt), several frameworks exist, which are described more in detail in Appendix A (Supplementary Materials).

Empirical evidence from experimental studies is needed to show differential effects of different kinds of adaptations (Heim et al., 2020). Using a standardised documentation system, such as proposed in this paper, is key to meta-analytic evidence that is based on high quality of research. To achieve this aim, it is vital to structure reports on cultural adaptations, and to enhance transparency on *what* was culturally adapted in psychological trials.

## Theoretical Framework

Heim and Kohrt (2019) propose a new framework of cultural adaptation that is based on evidence from cultural clinical psychology and psychotherapy research (see the section on Cultural adaptation frameworks in Appendix A, Supplementary Materials). The authors suggest using *cultural concepts of distress (CCD)* as the starting point for cultural adaptation. The term CCD has been introduced into the Diagnostic and Statistical Manual of Mental Disorders, fifth edition (DSM-5, American Psychiatric Association, 2013) to describe culturally shaped mental health-related phenomena. CCD encompass *idioms of distress* (Nichter, 1981, 2010), *cultural explanations* (Bhui & Bhugra, 2002), and *cultural syndromes* (Kaiser & Jo Weaver, 2019). Evidence shows that CCD differ from diagnostic categories in DSM and the International Classification of Diseases (Kohrt et al., 2014).

Heim and Kohrt (2019) further suggest using a taxonomy of treatment components proposed by Singla et al. (2017) to structure the cultural adaptation and reporting process. Different taxonomies to dismantle components of psychological interventions have been proposed in literature, e.g., for behaviour change interventions (Michie et al., 2013) or for interventions for children and adolescents (Chorpita & Daleiden, 2009). Based on such blueprints, Singla et al. (2017) proposed a taxonomy to distil the components of psychological interventions for the treatment of common mental disorders (i.e., depression, anxiety, and stress-related mental health issues) in low- and middle-income countries. This taxonomy consists of specific and nonspecific elements, and therapeutic techniques. Elements are therapeutic activities or strategies (e.g., problem solving), whereas techniques are skills that the therapist implements during a session (e.g., role-playing). Specific elements are grounded in specific psychological mechanisms (i.e., behavioural, cognitive, emotional, and interpersonal elements), and nonspecific elements are routed in common factors of psychological interventions (Cuijpers et al., 2019; Wampold, 2007).

Aside from elements and techniques, which refer to *what* is provided in treatment, Singla et al. (2017) describe the *how* (e.g., delivery format), *who* (e.g., non-specialists), *and where* (i.e., setting) of psychological interventions. In cultural adaptation, treatment aspects that are related to *how* content is transmitted, include, e.g., the consideration of different dialects in translation or culture-specific aspects in illustrations that are not directly related to therapeutic elements (e.g., Abi Ramia et al., 2018). In accordance with Resnicow et al. (1999), these are considered as adaptations of the surface (Heim & Kohrt, 2019).

## Process for Developing the Reporting Criteria

The Reporting Cultural Adaptation in Psychological Trials (RECAPT) criteria were developed by a “Task force for cultural adaptation of mental health interventions for refugees” in Germany. The aim of this task force was to harmonise and document the cultural adaptation process across eleven randomised controlled trials testing psychological interventions among refugees in Germany (Heim & Knaevelsrud, 2021, this issue). The task force developed a first set of criteria. Thereafter, an expert survey was conducted to seek consensus among international experts in the field of cultural adaptation and global mental health. Twenty-four international experts were invited, of which eleven responded to our survey and provided feedback on the reporting criteria. A second round of feedback was implemented, where the experts provided their comments on the revised criteria. For more details, please refer to Appendix A (Supplementary Materials). The expert survey is provided in Appendix B (Supplementary Materials).

## Reporting Criteria

In the following, we propose eleven reporting criteria for future psychological trials with different cultural and ethnic groups. Based on the theoretical and empirical considerations outlined above, the reporting criteria for bottom-up and top-down cultural adaptation in psychological trials are structured along the following categories: A) Set-up; B) Formative research methods; C) Intervention adaptation; D) Measuring outcomes and implementation. An overview of the eleven criteria is shown in Box 1. The last category, measuring outcomes, is kept short, as this is addressed in specific literature (e.g., Leong et al., 2019). However, because measuring outcomes is an integral part of randomised controlled trials, we decided to include it as part of the reporting criteria.

Box 1 **Reporting Cultural Adaptation in Psychological Trials (RECAPT): Overview of Criteria**

- Set-up
  Criterion 1: Definition of the target population
  Criterion 2: Team and roles
  Criterion 3: Documentation and monitoring system
  Criterion 4: Documentation of adaptations during trial (“on the fly”)
- Formative research
  Criterion 5: Formative research methods
  Criterion 6: Target symptoms, syndromes, needs, and context
- Intervention adaptation
  Criterion 7: Specific treatment elements
  Criterion 8: Nonspecific elements and therapeutic techniques
  Criterion 9: Surface adaptations
- Measuring outcomes and implementation
  Criterion 10: Questionnaires and clinical interviews
  Criterion 11: Implementation measures

We recommend reporting on these criteria, regardless of whether they were implemented or not. These reporting criteria can also be used as a guideline for planning the process of cultural adaptation of an existing intervention (top-down), or the consideration of cultural aspects in the development of new interventions (bottom-up) to be tested in psychological trials. The sequence of the reporting criteria is not fixed, as the process is often iterative; however, we put the sequence in what we considered to be a helpful order (e.g., establishing a documentation system early in the process). For reasons of word count, the description of each criterion is kept short. More detailed information can be found in the Appendix A (Supplementary Materials).

If possible, we recommend publishing a separate paper on formative research and cultural adaptation alongside the regular papers of a psychological trial (i.e., protocol and outcome paper), as it has been done in several studies (e.g., Abi Ramia et al., 2018). A separate paper allows researchers to provide detailed information on the decision-making process and the different adaptations that were implemented. If it is not possible to publish a separate paper on the formative research and cultural adaptation, it is still recommendable to report on the most important aspects in the protocol or results paper.

A reporting form that can be used for future trials is presented in the Supplementary Materials). For reasons of transparency and replicability, we recommend adding the documentation and monitoring sheet as supplementary material to published papers. The template is structured along the reporting criteria.

### A) Set-up

Cultural adaptation of psychological interventions is a complex process which most often includes several stages. Once a psychological trial is completed and results are about to be published, it may be difficult or impossible to reconstruct all the decisions that were made during the cultural adaptation process. For this reason, it is advisable to continuously document this process, and to be explicit about the people involved in decision-making.

#### Criterion 1: Definition of the Target Population

As described above, culture is a complex construct that cannot be reduced to ethnic groups or race. Many different socio-demographic factors may contribute to one’s “culture”, such as language, religion, age, migration background, refugee status, gender identity, sexual orientation, and socio-economic status, among others (González Castro et al., 2010; Sue & Sue, 2015). There is large variety with regard to values and norms within geographically or demographically defined groups (e.g., Fischer & Schwartz, 2011; Resnicow et al., 1999), and people may adopt different “cultural identities” in different contexts (Lehman et al., 2004).

Therefore, the first step in cultural adaptation is to clearly define the “unit of analysis”, i.e., the target population in the psychological trial (González Castro et al., 2010). The definition and operationalisation of this unit of analysis should be done along the most important criteria that may have an impact on participants’ cultural identity and their psychopathology (Betancourt & López, 1993). The unit of analysis may not always be limited to one particular ethnic, language, or even cultural group, i.e., psychological interventions can be culture-sensitive rather than culture-specific. Culture-sensitive interventions may target diverse groups, e.g., migrant populations in high-income countries, and be sensitive to cultural aspects in general rather than adapted to specific features of one particular group (e.g., Lotzin et al., 2021, this issue; Mewes et al., 2021, this issue).

#### Criterion 2: Team and Roles

Several guidelines for qualitative research (e.g., Malterud, 2001; Tong et al., 2007) consistently recommend providing information on the personal characteristics of the researchers involved in qualitative studies (e.g., occupation, gender, training and qualifications), as well as information about preconceptions, which represent previous experiences, pre-study beliefs, and motivation. In this sense, we recommend shortly describing the team that was involved in the cultural adaptation process, as well as their roles during the formative research phase and in the decision-making process.

#### Criterion 3: Documentation and Monitoring System

Documentation is key for transparency and replicability of clinical trials in general, and therefore also for the cultural adaptation process. When documenting the process of cultural adaptation, we suggest providing as much information as possible on CCD, on other relevant aspects in the target population (e.g., specific needs), on the foundations for decisions that were made (e.g., data gathered through focus group discussions), and on the strength of evidence to support such decisions.

Cultural adaptation most often starts with formative research (see below). In formative research, relevant information on the target population is gathered, and representatives of the target population are asked about the relevance and acceptability of the intervention. During this process, many suggestions for changing and adapting parts of the intervention may be made. Some of these suggestions may be absolutely essential, for instance because of ethical considerations, because not doing them may cause harm (e.g., stigmatization, hurting feelings of subgroups), or foster higher attrition rates. Moreover, a strong evidence-base might be a good indicator for the need of an adaptation. On the other hand, there may be changes that are “nice-to-have”, or even controversial, especially if they are based on personal preferences or taste (e.g., Shala et al., 2020).

#### Criterion 4: Documentation of Adaptations During Trials (“On the Fly”)

In most running trials, some level of adaptation may happen “on the fly”, especially when working with diverse ethnic and cultural groups, for whom we have less empirical evidence on psychological interventions (Unterhitzenberger et al., 2021, this issue). As an example, if a misunderstanding in psychoeducation is discovered, it might be necessary to adapt the wording and, if needed, provide standard translations of such psychoeducation to interpreters for the rest of the trial. One may argue that ideally, such difficulties are discovered in pilot trials that are done exactly for this purpose. However, it is still possible that important information is revealed in the course of running trials, and documentation and transparency with regard to such “on-the-fly” adaptations may be relevant for a better understanding of trial results and implementation.

In this line of thinking, Chambers and Norton (2016) challenge the assumption of a linear, static process from intervention development (and adaptation) to pilot testing, randomised controlled trial, and implementation. In this linear view that is still prevailing in literature, deviances from manuals are considered to be problematic, as they may threaten treatment fidelity and thus, effectiveness of the intervention. In their publication entitled “The Adaptome - Advancing the Science of Intervention Adaptation”, Chambers and Norton (2016) aim to capture “positive deviance (e.g., where adaptation leads to better outcomes compared to the original trials) as well as circumstances in which program drift was deleterious to intervention effectiveness” (p. 127). Thus, Chambers and colleagues make a case for documenting deviances from originally defined protocols: “By augmenting trial data with practice-based evidence, we can understand much more about what works for whom” (Chambers et al., 2013, p. 6). Using a standard documentation system (RECAPT Template, Supplementary Materials) will enhance transparency on adaptations that were made during trials.

### B) Formative Research

Formative research includes the iterative process of gathering relevant information before starting a trial. The process of formative research is ideally reported in a consistent and transparent manner, to ensure replicability and valid interpretation of results. The RECAPT criteria include the *methods* of formative research on the one hand, and the *results* of this process on the other hand.

#### Criterion 5: Formative Research Methods

Formative research is an iterative process using multiple qualitative and quantitative methods. In the following, we provide suggestions on how to implement this process, thus, on *how to adapt*. In the Supplementary Materials, we provide a Template for documenting the cultural adaptation process. Formative research methods (i.e., literature review, qualitative, quantitative, and mixed methods) can be flexibly used until a level of saturation is reached. Results of this process should highlight the description of the target population’s main characteristics, their most salient symptoms or syndromes and needs, and the feedback gathered on the intervention during the process of cultural adaptation. Although there is no “standard procedure” for top-down or bottom-up cultural adaptation, we suggest reporting on these different stages of formative research.

Formative research normally starts with a *literature review*. Thereafter, researchers may conclude that available evidence on their target population is insufficient for cultural adaptation. *Qualitative and/or quantitative information* on the target population (i.e., main characteristics, symptoms, syndromes, needs) should be gathered where no or insufficient evidence is available, including mixed methods approaches (Shala et al., 2020; Singla et al., 2014). Quantitative methods include symptoms scales, surveys, or other questionnaires used to describe the target population. Qualitative methods include in-depth interviews with key informants, focus groups, free-list interviews, pile sorting, among others (Cork et al., 2019; Keys et al., 2012).

We recommend using the consolidated criteria for reporting qualitative research (COREQ, Tong et al., 2007), a 32-items checklist for explicit and comprehensive reporting of qualitative studies. It includes participant selection (i.e., selection, method of approach, sample size, reasons for refusing); the setting for data collection (e.g., home, clinic); the method of data collection (i.e., interview guide, recording, duration), and the analysis methods (i.e., how themes were derived from the data).

Once data on the target population is gathered and compiled, interventions are adapted in a bottom-up or top-down approach. This process is accompanied by formative research, as well. And iterative process of adaptation, validation, and piloting is recommended (e.g., Shala et al., 2020). Regardless of the methods chosen in the process of cultural adaptation, documentation is key.

#### Criterion 6: Target Symptoms, Syndromes, Needs, and Context

This criterion describes the most relevant aspects to consider in cultural adaptation. As outlined above, Heim and Kohrt (2019) suggest using CCD as the pivotal point for cultural adaptation. CCD are distinct from diagnostic categories such as depression, or post-traumatic stress, but in many cases share symptoms with these disorders (e.g., Haroz et al., 2017; Rasmussen et al., 2014). Examples of CCD in literature are spirit possession in Uganda and Zimbabwe (Ertl et al., 2011; Patel et al., 1995), *dhat* in India (i.e., semen loss in urine; Gautham et al., 2008), *hwa-byung* in Korea (i.e., fire/projection of [accumulated] anger into the body; Min & Suh, 2010), or *khyâl attacks* (i.e., wind attacks) in Cambodia (Hinton et al., 2010). Evidence shows that CCD are often associated with symptoms of psychological distress and mental disorders in general. However, it would be erroneous to conclude that CCD are just variations of the same (universal) underlying constructs across cultural groups. In their systematic review on CCD, Kohrt et al. (2014) argue that higher methodological rigour is needed to better understand potential associations and distinctions between CCD and diagnostic categories developed in Western countries. We recommend using an ethnopsychological model to frame the understanding and use of CCDs (Keys et al., 2012; Kohrt & Hruschka, 2010).

Other relevant topics for cultural adaptation may include specific needs in the target population, mental health related stigma, as well as contextual variables such as differential exposure to social determinants of mental health, and access to health systems, and mental health resources (Hook et al., 2021). An example of such a contextual variable is ongoing armed conflict, which requires specific contextual adaptation of psychological interventions (Castro-Camacho et al., 2019).

### C) Intervention

In clinical and empirical literature, cultural adaptation of psychological interventions most often implicitly refers to the top-down approach, in which existing psychological interventions developed for one cultural group are adapted for another one (Hall et al., 2016; Hwang, 2006). There is little evidence on psychological interventions adapted in a bottom-up approach to address culture-specific symptoms and syndromes.

One might argue that the development of new interventions does not fall under “adaptation”. We counter this argument by stating that psychological interventions for the treatment of distress and mental disorders are a “Western” concept by themselves, as is the empirical evaluation of such interventions through randomised controlled trials. Therefore, the present reporting criteria are applicable not only for trials testing culturally adapted versions of existing interventions, but also newly developed interventions and intervention components that aim to target specific factors among culturally diverse groups.

Psychological interventions and trials to evaluate them share a common set of features, which have been classified by Singla et al. (2017) into four categories: Who (i.e., provider); What (i.e., treatment components); Where (i.e., treatment setting); and How (i.e., training, supervision, treatment delivery). Treatment components can be distilled into i) specific elements that are based on theoretical psychological models; ii) nonspecific elements that are commonly shared by interventions of different theoretical backgrounds; and iii) therapeutic techniques that aim to transmit specific and nonspecific elements (see Theoretical framework above). This taxonomy provides a helpful grid to support the cultural adaptation of intervention, as it specifies the different levels of an intervention. Other frameworks (e.g., Bernal et al., 1995; Bernal & Sáez-Santiago, 2006) have listed elements for cultural adaptation without putting them into a functional relationship. Accordingly, we structured our reporting criteria along the taxonomy by Singla et al. (2017).

The template provided in the Supplementary Materials can be used for documenting cultural and contextual adaptations, evidence to support each decision, and suggestions from the research team.

#### Criterion 7: Specific Treatment Elements

Most psychological trials have used manuals or protocols as unit of analysis (Chorpita & Daleiden, 2009). Manuals most often focus on one particular diagnosis and use a series of elements for the treatment of this disorder (e.g., psychoeducation, exposition, cognitive restructuring, relapse prevention) for their treatment. Transdiagnostic interventions combine treatment elements to address a broader symptom spectrum instead of one particular diagnosis, with promising effect sizes (Newby et al., 2013). As an example, the Common Elements Treatment Approach (CETA, Murray et al., 2014), applies evidence-based treatment elements depending on the specific symptomatology of the patient. Other examples are Problem Management Plus (PM+, Dawson et al., 2015) developed by World Health Organization (WHO), or the Unified Protocol for Emotional Disorders (Barlow et al., 2004).

As mentioned in the Theoretical framework (see above), single treatment components can be distilled from such manuals, and several authors advocate reporting on treatment components (rather than manuals) in randomised controlled trials. In the process of cultural adaptation, this distillation may be even more relevant. In psychological trials with diverse ethnic and cultural groups, it may be important to provide some empirically or theoretically based rationale for the selection, omission or adaptation of each of the specific treatment elements. In addition, explicit decisions to leave specific elements unchanged should be reported, as well (Böttche et al., 2021, this issue).

The mental health Cultural Adaptation and Contextualization for Implementation (mhCACI) procedure begins with identification of the mechanisms of action as the first step in order to inform the literature review, formative work, and other steps (Sangraula et al., 2021). The literature review and formative work can be used to determine which specific treatment elements and other mechanisms of action will best fit with the culture and context. Alternatively, the literature review and formative work can be used to select which type of intervention will fit best and is mostly likely to undergo successful adaptation. If the CCD, community needs, and context are clearly defined, this will inform which interventions would not require heavy adaptation for implementation, which is especially important when rapid deployment is needed such as during humanitarian emergencies.

#### Criterion 8: Nonspecific Elements and Therapeutic Techniques

Nonspecific elements refer to components that are universal to all treatments, also known as “common factors” (Cuijpers et al., 2019; Wampold, 2007). One important common factor is the provision of a convincing treatment rationale. Psychological interventions ideally provide explanations that differ from the patient’s views, but that are not too discrepant from the patient’s intuitive assumptions as to be rejected (Wampold, 2007). This suggests trying to find common ground between the treatment’s hypothesized mechanism of action (including both specific and nonspecific elements) and the patient’s explanatory model. For treatment adherence and compliance, it is vital that patients understand and to some point share the rationale behind the treatment. The treatment rationale is ideally dovetailed with cultural explanations and idioms of distress that are part of CCD (Hwang, 2006; Rathod et al., 2019). CCD may include beliefs and assumptions that require to be challenged when providing the treatment rationale.

In addition, it may be relevant to consider culture-specific notions of stigma, and the way how mental health-related stigma threatens the life domains that “matter most” (Yang et al., 2014) to members of a specific cultural group (e.g., marriage, employment, social networks). Intervention adaptation should include consideration of "what matters most" because this will influence stigma and motivation of those delivering the intervention (Kohrt, Turner, et al., 2020). Documentation of how adaptations address what matters most further demonstrates the rigor of the approach.

We also recommend to report on the reflections that have guided the choice, omission, or adaptation of *therapeutic techniques*, such as role-playing, goal setting, or homework (Singla et al., 2017).

#### Criterion 9: Surface Adaptations

Surface structure adaptations aim to enhance acceptability of an intervention through matching materials, channels and settings to the target population (Resnicow et al., 1999). Such surface adaptations correspond to the *How* and *Where* in the taxonomy suggested by Singla et al. (2017). There is much evidence on such surface adaptations of psychological interventions (Chowdhary et al., 2014; Chu & Leino, 2017; Harper Shehadeh et al., 2016).

Cultural and contextual factors may determine the channels through which the treatment components are provided, e.g. group-based as opposed to individual treatment (Epping‐Jordan et al., 2016; Sangraula et al., 2018; Verdeli et al., 2003), or internet-based interventions (Naslund et al., 2017) that are increasingly tested and applied among diverse ethnic and cultural groups. Reporting should include considerations that have been made with regard to such different modes of delivery.

Interventions (both self-help and face-to-face) may include materials such as texts, illustrations, case examples, flyers, audio files, videos, etc. Standards exist for the translation of assessments and materials (e.g., van Ommeren et al., 1999). Several studies report that it is often difficult to draw the line between translation and adaptation, as these two are closely intertwined (Ramaiya et al., 2017; Shala et al., 2020). For pragmatic reasons, it is often not possible to document all the decisions that were made during the process of translation and language editing, especially if the decisions are merely questions of style or grammar. However, some decisions might be relevant to be documented in the cultural adaptation monitoring sheet. As an example, metaphors are often culture-specific and cannot be translated literally (Rechsteiner et al., 2020). It might therefore make sense to report on how specific metaphors in the intervention were translated or adapted.

### D) Measuring Outcomes and Implementation

As outlined above, there is considerable cultural variation in symptom expression. In clinical trials testing psychological interventions among diverse ethnic and cultural groups, it is important to account for this cultural validation by using validated instruments.

#### Criterion 10: Questionnaires and Clinical Interviews

When conducting clinical trials with culturally diverse populations, it is vital to provide information on the extent to which outcome measures (i.e., questionnaires and clinical interviews) were translated, (culturally) adapted, and validated.

There are standard criteria for the translation, adaptation, and validation of questionnaires. As an example, Wild et al. (2005) and van Ommeren et al. (1999) provided principles of good practice for the translation and cultural adaptation process for patient-reported outcomes. In addition, standard psychometric methods for the cross-cultural validation of questionnaires and measurement invariance have been developed (e.g., Byrne et al., 1989; Chen, 2008; Milfont & Fischer, 2010; Vandenberg & Lance, 2000). Several standard questionnaires have been used for application among diverse cultural and ethnic groups, e.g., the Patient Health Questionnaire (Kroenke & Spitzer, 2002), the Generalised Anxiety Disorder scale (Spitzer et al., 2006), the Posttraumatic Diagnostic Scale (Foa et al., 1997), the General Health Questionnaire (Goldberg, 1972), or the WHO Disability Assessment Scale (Ustun et al., 2010), to mention only a few.

The validity of questionnaires can be enhanced by incorporating CCD, and particularly idioms of distress. Another option is the use of client-generated outcome measures, such as the Psychological Outcome Profiles instrument (PSYCHLOPS, Ashworth et al., 2004), which has been validated in several countries (e.g., Czachowski et al., 2011; Héðinsson et al., 2013). Another client-generated outcome measure is the Personal Questionnaire (Elliott et al., 2016).

Most trials use self-report questionnaires as their primary outcome measure. Clinical interviews are of course more labour-intensive, but the diagnostic accuracy might be higher (Ferrari et al., 2013), especially among diverse cultural and ethnic groups. If the planned outcome measure for the psychological trial is a clinical interview (e.g., the Structured Clinical Interview for DSM-5, SCID-5-CV; First et al., 2016), it is recommended to integrate a culture-sensitive interview, such as the *Cultural Formulation Interview* in DSM-5 (American Psychiatric Association, 2013). Training interviewers in culture-sensitive assessments is important, in order to avoid misdiagnosis. And it is relevant to report on interviewer training and interrater reliability with regard to cultural competence.

#### Criteria 11: Implementation Measures

In addition to measuring outcomes, the implementation process should be documented, as well. Without documenting implementation, it is difficult to determine if an unsuccessful intervention is due to the intervention not being effective or lack of fidelity when delivering the intervention (Jordans & Kohrt, 2020; Kohrt, El Chammay, et al., 2020). Moreover, assessing implementation is vital to determine that the cultural adaptations were actually enacted in delivery of the intervention. Criterion 11 refers to the *Who* and *How* criteria in the taxonomy by Singla et al. (2017).

In addition to fidelity, competency of providers is important to evaluate. Competency tools now exist that can be modified based by the culture and context for a psychological intervention (Kohrt, Schafer, et al., 2020); these address both competency in nonspecific treatment factors (Kohrt et al., 2015) and culturally adapted competencies in treatment specific factors, such as for PM+ (Pedersen et al., in press).

### Quality Rating

Currently there are no standards for ranking of cultural adaptation quality. We propose for preliminary use that cultural adaptation studies that only report on 4 or fewer of the criteria be consider ‘low quality’ of reporting. Studies that are 5-8 criteria be identified as ‘moderate quality’ of reporting. Finally, studies that clearly document 9-11 criteria be considered ‘high quality’. These rankings are subject to change as more documentation occurs on adaptation and further research is conducted about what aspects of adaptation matter most for successfully alleviating suffering across cultures and context around the world.

## Concluding Remarks

In this paper, we propose a set of reporting criteria for cultural adaptation in clinical trials which test psychological interventions among diverse cultural and ethnic groups. Although these reporting criteria were primarily developed for treatments of common mental disorders, they may be used also for other kinds of interventions, such as prevention or mental health promotion.

The suggested set of criteria was compiled based on the authors’ experiences and current literature. Although not exhaustive, the criteria are comprehensive and may be used for top-down and bottom-up cultural adaptation (Hall et al., 2016; Hwang, 2006). They can be used to guide the process of cultural adaptation, as well as for documentation. That said, it is likely that not all of these criteria are relevant for all trials conducted in this field of research. In this sense, the use and the sequence can be adapted flexibly to the needs of researchers. A template for documenting the process and results of cultural adaptation can be found in the Supplementary Materials.

Reporting on cultural adaptation more consistently in future psychological trials will hopefully improve the quality of evidence and contribute to examining the effect of cultural adaptation on treatment efficacy, feasibility, and acceptability.

## Supplementary Materials

The Supplementary Materials contain the following items (for access see Index of Supplementary Materials below):

- **Appendices**
  - *Appendix A* provides additional information on definitions of culture, cultural adaptation literature, the process for developing the RECAPT criteria, and detailed information on each criterion.
  - *Appendix B* shows the expert survey used for developing the RECAPT criteria.
- **RECAPT Template**
  A template for documenting the cultural adaptation process that was developed by the “Task force for cultural adaptation of mental health interventions for refugees”. A documented version for better understanding is provided, along with an empty template in Word format that can be used for future studies.

10.23668/psycharchives.5201 Supplement 1 Supplementary materials to "Reporting Cultural Adaptation in Psychological Trials – The RECAPT criteria" [Appendices]

10.23668/psycharchives.5192 Supplement 2 Supplementary materials to "Reporting Cultural Adaptation in Psychological Trials – The RECAPT criteria" [RECAPT Template]

HeimE.
MewesR.
Abi RamiaJ.
GlaesmerH.
HallB.
Harper ShehadehM.
ÜnlüB.
KananianS.
KohrtB. A.
Lechner-MeichsnerF.
LotzinA.
MoroM. R.
RadjackR.
Salamanca-SanabriaA.
SinglaD. R.
StarckA.
SturmG.
TolW.
WeiseC.
KnaevelsrudC.
 (2021a). Supplementary materials to "Reporting Cultural Adaptation in Psychological Trials – The RECAPT criteria"
[Appendices]. PsychOpen. 10.23668/psycharchives.5201
PMC967082636405678

HeimE.
MewesR.
Abi RamiaJ.
GlaesmerH.
HallB.
Harper ShehadehM.
ÜnlüB.
KananianS.
KohrtB. A.
Lechner-MeichsnerF.
LotzinA.
MoroM. R.
RadjackR.
Salamanca-SanabriaA.
SinglaD. R.
StarckA.
SturmG.
TolW.
WeiseC.
KnaevelsrudC.
 (2021b). Supplementary materials to "Reporting Cultural Adaptation in Psychological Trials – The RECAPT criteria"
[RECAPT Template]. PsychOpen. 10.23668/psycharchives.5192
PMC967082636405678
