# Supplementary material for: Reporting Cultural Adaptation in Psychological Trials – The RECAPT criteria
Source: Clin Psychol Eur. 2021 Nov 23;3(Spec Issue):e6351. doi: 10.32872/cpe.6351 (PMC9670826; doi:10.32872/cpe.6351)
Supplement: Supplement 1 [file cpe-03-6351-s01.pdf]

Supplementary Materials

1) Appendix A: Additional Information
2) Appendix B: Expert survey

Appendix A: Additional information

1 Definitions of culture 2

2 Cultural adaptation frameworks 2

3 Process for developing the RECAPT criteria 3

4 Detailed information on the RECAPT criteria 4

4.1 Definition of the target population 4

4.2 Team and roles 5

4.3 Documentation and monitoring system 5

4.4 Documentation of adaptations during trials (“on the fly”) 6

4.5 Formative research methods 6

4.6 Target symptoms, syndromes, needs, and context 7

4.7 Specific treatment elements 8

4.8 Nonspecific elements and therapeutic techniques 8

4.9 Surface adaptations 9

4.10 Questionnaires and clinical interviews 9

4.11 Implementation measures 9

1 Definitions of culture

In the *Lancet Commission on Culture and Health*, culture is defined as follows: "Culture, then, can be thought of as a set of practices and behaviours defined by customs, habits, language, and geography that groups of individuals share" (Napier et al., 2014, p. 1609). The Commission further points out,

> "Culture… cannot be merely equated with ethnic group or national allegiance. We all participate in locally defined forms of behaviour that not only produce social cohesion, but that limit our ability to see the subjective nature of our values, our perceived responsibilities, and our assumptions about objective knowledge. In this context, the responsibilities of doctors and health systems, and the priorities of policy makers and researchers, are also collective behaviours based on social agreements and assumptions—i.e., on culture" (p. 1608).

The Cultural Framework for Health produced by the U.S. National Institutes of Health includes the following definition:

> "Culture consists of dynamic and ecologically based inter-related elements that function together as a living, adapting system. To delineate culture begins with a perspective that contextualizes population groups within a multi-level, multi-dimensional, biopsychosocial, ecological framework and explicitly recognizes and incorporates the geographic, historical, social, and political realities of diverse communities. All of these elements constitute the cultural framework its members use to "see" the world and attribute meaning to their daily lives" (Kagawa Singer et al., 2015, p. 12)

2 Cultural adaptation frameworks

The most prominent framework was developed by Bernal and colleagues (Bernal et al., 1995; Bernal & Sáez-Santiago, 2006). This heuristic framework encompasses eight treatment elements to be considered in cultural adaptation (i.e., language, therapeutic relationship, metaphors, content, methods, concepts, goals, and context). Bernal and colleagues proposed these elements without specifying their relative importance or effect.

Resnicow et al. (1999) differentiate between surface and deep structure adaptations of evidence-based interventions in health promotion and prevention. According to the authors, surface structure adaptations foster the acceptance and feasibility of an intervention by matching materials (e.g., illustrations, language), as well as channels and settings for treatment delivery to observable characteristics of the target population. By contrast, deep structure adaptations take into account how cultural, social, environmental, or historical factors influence health behaviours. Such adaptations are based on assumptions of how members of a particular cultural group perceive the

cause, course, and treatment of a particular illness. Deep structure adaptations convey salience of an intervention and determine its efficacy or impact.

More recently, Chu and Leino (2017) developed a similar framework in which they differentiate between adaptations of *peripheral* vs. *core* elements in psychological treatments. Peripheral aspects correspond to what Resnicow et al. (1999) describe as surface, thus all aspects related to feasibility and acceptance. Core elements are the therapeutic ingredients that are assumed to cause symptom change, based on psychological theories. The division into core and peripheral aspects is based on the assumption that the mechanisms of action in psychological interventions are known. However, two prominent systematic reviews conclude that current evidence is insufficient to explain change mechanisms in psychotherapy (Cuijpers et al., 2017; Lemmens et al., 2016). Aside from specific core interventions, common factors such as the therapeutic alliance, positive expectations, and a convincing treatment rationale most likely contribute to treatment efficacy (Cuijpers et al., 2019; Wampold, 2007, 2015). Thus, what Chu and Leino (2017) consider as peripheral adaptations might actually contribute to such common factors, thereby enhancing efficacy alongside feasibility and acceptance.

In summary, the existing frameworks for cultural adaptation have provided important contributions, but they also have shortcomings. Bernal and colleagues (Bernal et al., 1995; Bernal & Sáez-Santiago, 2006) put together the relevant elements for cultural adaptation, but without specifying how these elements relate to one another. Resnicow et al. (1999) and Chu and Leino (2017) use different lines of argument to make a distinction between adaptations that foster treatment *feasibility / acceptance* vs. *efficacy*, without referring to the current status of psychotherapy research on mechanisms of action. A theory-driven framework for cultural adaptation of psychological interventions in clinical trials ideally includes the relevant elements of cultural adaptation without making pre-assumptions on whether these elements are related to treatment feasibility, efficacy, or both.

3 Process for developing the RECAPT criteria

In 2016, the German Federal Ministry of Education and Research launched a call for research proposals on “Mental health of refugee populations”. As a consequence, a variety of psychological interventions for the treatment of common mental disorders are currently being tested in eleven randomised controlled trials among diverse groups of displaced people in Germany. In addition, a “Task force for cultural adaptation of mental health interventions for refugees” was established. The aim of this task force, which consists of representatives of the eleven randomised controlled trials,

was to harmonise and document the cultural adaptation process across studies. The aims and work of this task force are described more in detail elsewhere (Heim & Knaevelsrud, 2021).

Based on the collected experiences, a sub-group of the task force developed the present reporting criteria (EH, RM, HG, SK, FLM, AL, AS, CW, CK). Thereafter, an online survey with international experts in cultural adaptation of psychological interventions was conducted. Experts were selected based on their contributions to cultural adaptation literature, and they were asked to provide names of other experts.

The online survey included two rounds of feedback. In the first round, experts were asked to provide feedback on the criteria. They indicated their general agreement with each criterion and with the corresponding descriptions. In addition, they rated the relevance and the applicability of each criterion on a 4-point scale. They were also asked to provide additional feedback in an open-ended text field. At the end of the survey, the experts were asked to suggest further criteria, and other experts to be invited for the survey (Appendix B). The reporting criteria were adapted based on the feedback provided by these experts. (The detailed feedback can be provided by the authors upon request). Thereafter, a second round of feedback was done, where the experts were invited to provide comments on the revised version of the criteria. The final manuscript was sent to all experts for a last review. A total of 24 international experts were invited to participate in the survey, of which eleven responded to the survey and provided feedback. All experts who provided feedback were listed as co-authors of the RECAPT criteria (JAB, BH, MH, BIU, BK, MRM, RR, ASS, DRS, GS, WT).

The "Task force for cultural adaptions of mental health interventions for refugees" (Heim & Knaevelsrud, 2021) has also developed a template for documenting cultural adaptations across a series of psychological trials with refugee populations in Germany (Heim et al., 2021). This template was based on earlier work by Shala, Morina, Burchert, et al. (2020) and inspired by a translation monitoring form (van Ommeren et al., 1999). An example of a completed template can be found in Lotzin et al. (2021). The experiences in the task force showed that the form was overly complex, as it included several sub-sections that were difficult to differentiate. The form was further adapted based on these experiences. The international experts who had responded to the survey were asked to provide feedback on the template, as well, along with the final manuscript.

4 Detailed information on the RECAPT criteria

4.1 Definition of the target population

For others to judge the generalizability of a cultural adaptation, the language(s) and any information on particular dialects should be clearly documented. If an adaptation was done in Arabic, was it done with Arabic speakers in Lebanon, Tunisia, Sudan, or another region. The age range addressed by the

adaptation is important because of major intergenerational differences in mental health concepts. Therefore, is an adaptation intended to address older adults, middle-aged adults, youth, or children, or is there only target group, e.g., senior adult members of a group. This will inform who should be part of the teams and roles, as well the focus of the formative research. The economic status of the target population should be defined. An adaptation in which formative work is only conducted with middle- and upper- class members of a society may have significant shortcomings when delivered to economically marginalized groups. Populations defined by their legal status and residential stability will require particular adaptations, therefore, it is important to clarify if the group is refugees, undocumented populations, internally displaced groups, etc. In summary, we strongly recommend clear documentation of the intended population for adaptation so that others can judge whether the adaptation process was consistent with this group and to what degree the adaptations can be generalized to other populations and settings.

4.2 Team and roles

As Malterud (2001) outlines, providing information of the team and roles contributes to the process of reflexivity and accounts for the view that knowledge is partial and situated. Providing information about the researchers' background and preconceptions may contribute to the reflection about what aspects in the encounter between researchers and study participants may have contributed to or shaped the results, and therefore reduce bias.

This information can include details related to fluency in the target language for adaptation, residence or background similar to population of the intended adaptation. The cultural adaptation of an online self-help intervention for overseas Filipino workers in Macau (Garabiles et al., 2019) provides an example. With increasing attention to patient and public involvement (PPI) in research (Boivin et al., 2018; Price et al., 2018), this section should also address people with lived experience of mental illness from the cultural group who are part of the adaptation team.

4.3 Documentation and monitoring system

When documenting the process of cultural adaptation, we suggest providing as much information as possible on cultural concepts of distress, on other relevant aspects in the target population (e.g., specific needs), on the foundations for decisions that were made (e.g., data gathered through focus group discussions), and on the strength of evidence to support such decisions. A central component is describing ethnopsychological or ethnophysiological models of distress. Ethnopsychology frames how distress is experienced in relation to concepts of the mind, the body, the soul, social relations, and other aspects of the self (Kohrt & Hruschka, 2010; White, 1992). Ethnopsychology of the intended populations because these models influence how the intervention is delivered and its

impact (Hinton & Otto, 2006; Kohrt et al., 2012). For example, ethnopsychological models may lead to the same intervention being interpreted and enacted differently by clinicians and beneficiaries.

If particular aspects are mentioned by different key informants or brought up consistently across different focus groups, this may be strong evidence for a necessary adaptation. By contrast, if a suggestion is based on one individual opinion, this is considered to be weak evidence. Based on the chosen methods of formative research, it may be recommendable to pre-define criteria for decision-making and the required strength of evidence for such decisions. Since most of the times not all adaptions can be made in the very beginning, the system should also have a section for documenting adaptions later on.

4.4 Documentation of adaptations during trials ("on the fly")

As outlined in the main document, some level of adaptation may happen "on the fly" in running trials. In case of a multi-centre trial, it is important to decide how relevant information on such on-the-fly adaptations should be reported back to principal investigators, and who is entitled to make decisions on such adaptations. It is thus recommendable to have a system in place for decision-making and documentation of such deviations.

Due to the current procedures with ethical protocols and trial registration, in which all relevant changes have to be approved by ethical review committees in amendments to protocols, researchers might be hesitant to document changes in procedures or interventions in running trials. We recommend using a documentation and monitoring sheet (see above) and add this information as supplementary material to the published paper on the main results of the trial, or the separate paper on cultural adaptation.

4.5 Formative research methods

Literature review. In their framework for the cultural adaptation of low-intensity psychological interventions in humanitarian settings, Perera et al. (2020) suggest performing a *desk review.* According to Greene et al. (2017) "[d]esk reviews are intended to pragmatically synthesize academic and grey literature to deliver timely information in a readable style for practitioners" (p. 2). In humanitarian situations, where a response needs to be provided immediately, it makes sense to speed up this process. By contrast, when testing new interventions in psychological trials without imminent urgency, the process of literature review may take more time. Existing literature (including grey literature) on symptoms, syndromes and needs in the target population is needed as a starting point for bottom-up and top-down adaptation. In addition, it is relevant to identify literature on the cultural adaptation of psychological interventions for specific target groups, e.g. as done for Arab countries (Gearing et al., 2012).

Qualitative and quantitative research methods. Participatory action research (PAR) and community based participatory research (CBPR) are ideal methodologies with associated tools for beneficiaries to guide the formative research process and associated adaptations (Collins et al., 2018; Dickerson et al., 2020; Walters et al., 2020). Therefore, any use of these techniques with community engagement to judge the quality of the adaptation are helpful.

Applying a multi-stakeholder perspective is recommended, including clinicians, community members, potential users of the intervention, and other relevant groups. Cork et al. (2019) found that in most studies, interviews were done with community samples and not with patients. However, for assessing needs in the target population and particularly CCD (see Criterion 6), it is vital to conduct interviews with a clinical sample, to gather their own symptom expressions and cultural explanations (i.e., etiological assumptions).

There are several instruments to examine CCD in clinical samples, e.g., the *Cultural Formulation Interview* in DSM-5 (American Psychiatric Association, 2013), the *Short Explanatory Model Interview* (SEMI, Lloyd et al., 1998)*, the Barts Explanatory Model Inventory* (BEMI, Rüdell et al., 2009) or the *McGill Illness Narrative Interview* (MINI, Groleau et al., 2006). These interviews cover idioms of distress, specific target symptoms (e.g., anger, pain, or dizziness), cultural explanations (i.e., explanatory models), culture-specific syndromes, beliefs about the course of the disorder and help-seeking behaviour. Depending on the available time, the use of such standardised interviews is recommended.

Once data on the target population is gathered and compiled, interventions are adapted in a bottom-up or top-down approach. This process is accompanied by formative research, as well. An iterative process of adaptation, validation and piloting is recommended (e.g., Shala, Morina, Burchert, et al., 2020). Studies on cultural adaptation of psychological interventions have used focus groups and key informant interviews during this process (Abi Ramia et al., 2018; Garabiles et al., 2019). In addition, “cognitive interviewing” (Willis, 2004) has been used frequently in literature, in which intervention materials are read through and participants “think aloud”. Wingood and DiClemente (2008) suggest identifying “topical experts” who can assist in adaptation. And Salamanca-Sanabria et al. (2019) developed an instrument, the Cultural Relevance Questionnaire (CRQ) to support and document the cultural adaptation of psychological interventions. Regardless of the methods chosen in the process of cultural adaptation, documentation is key. Results can be documented in the cultural adaptation monitoring form (Heim et al., 2021).

4.6 Target symptoms, syndromes, needs, and context

Empirical evidence on CCD is emerging in some parts of the world (e.g., Africa, Latin America, East Asia; Kohrt et al., 2014), but there is still a considerable lack of evidence from other regions, e.g.

South-Eastern Europe (Shala, Morina, Salis Gross, et al., 2020). It is important that CCDs are not mere 'vocabulary lists' that are devoid of a conceptual framework to understand how they are connected to one another and the relation to other understandings of the self, suffering, social behaviour, etc. Therefore, as mentioned above, we recommend using an ethnopsychological model to frame the understanding and use of CCDs (Keys et al., 2012; Kohrt & Hruschka, 2010). This is especially helpful when trying to determine which CCDs are particularly stigmatized. Documentation of adaptation should thus capture the ethnopsychological framework in addition to specific CCDs.

4.7 Specific treatment elements

Specific treatment elements, even if evidence-based, might sometimes not or only partly correspond to the most salient symptoms in the target group. As an example, behavioural activation is based on the theoretical assumption that inertia and avoidance are important maintaining factors in depression (Ferster, 1973; Lewinsohn, 1974; Veale, 2008), and behavioural activation a key mechanism of action in depression treatment. However, a systematic review of qualitative studies about depression around the world demonstrated that "the majority of study populations did not raise problems with daily functioning as part of their subjective experiences of depression" (Haroz et al., 2017, p. 160). Instead, irritability, anger, and pain were prominent symptoms often related to depression. This raises the question whether behavioural activation is the first choice as a specific element to treat depression among diverse cultural and ethnic groups, or whether other interventions, e.g., stress management and emotion regulation skills, would be more effective.

Studies with diverse ethnic and cultural groups sometimes provide reasons for the choice of specific components, e.g. arguing that behavioural activation is easier to explain than cognitive techniques, especially if the intervention is provided by lay helpers (Dawson et al., 2015). Two studies in this issue (Böttche et al., 2021; Kananian et al., 2021) showed their reasons for choosing/omitting/adapting problem management as specific treatment elements in their interventions with refugees. Similarly, Carswell et al. (2018) developed a mobile- and Internet-based intervention (called Step-by-Step) for the treatment of depression among populations affected by adversities in low- and middle-income countries. Although Step-by-Step was based on the manual called Problem Management Plus (Dawson et al., 2015), problem management was omitted from the Step-by-Step intervention because it was assumed to be difficult to explain through a self-help intervention provided through technology.

4.8 Nonspecific elements and therapeutic techniques

Cork et al. (2019) conducted a systematic review on how to integrate CCD, and particularly idioms of distress, into existing mental health assessments and interventions. CCD may include beliefs and assumptions that require to be challenged when providing the treatment rationale. As an example,

Reich et al. (in press) developed a web-based intervention to enhance motivation for psychotherapy among Turkish immigrants in Germany who held fatalistic beliefs.

Reporting on cultural adaptation of nonspecific components is recommended, especially to enhance empirical evidence on the effect of this adaptation. As an example, if the treatment rationale is culturally adapted, while leaving all other components of the intervention unchanged, this may indicate that the rationale itself is a mechanism of action in the corresponding treatment (Heim et al., 2020).

4.9 Surface adaptations

For self-help interventions such as internet- and smartphone-based programmes, an additional process is required of creating software functions and user-interfaces that match the chosen content and therapeutic techniques as well as characteristics of the intended users. Ideally, this process is conducted in a systematic manner using design thinking and user-centred design approaches (Mummah et al., 2016). These approaches utilize interviews, focus groups, mock-ups, and interactive prototypes in an iterative manner to optimize usability and the fit with the needs, expectations, and technical proficiency of the target group (e.g., Burchert et al., 2019).

4.10 Questionnaires and clinical interviews

The validity of questionnaires can be enhanced by incorporating CCD, and particularly idioms of distress. As an example, Hinton et al. (2019) suggest adding items such as ‘‘thinking a lot,’’ ‘‘weak heart,’’ or ‘‘blurry vision’’ to the assessments of depression and anxiety among Cambodian patients, as these symptoms are salient for psychological distress in this particular group. In this case there is a core instrument that is still comparable across different groups (if measurement invariance has been established), but specific idioms of distress are considered to establish a culture sensitive part of the assessment. In their systematic review, Cork et al. (2019) found that some studies integrated idioms of distress into existing questionnaires while others developed new assessments for the groups where CCD were gathered. Newly developed culture-specific instruments may thus capture the symptomatology in this specific group, but they impede cross-cultural comparisons. The strategy of integration of idioms of distress thus depends on context and aims of the diagnostic assessments.

4.11 Implementation measures

There are a range of tools and domains that should be assessed for implementation. Fidelity to the intervention is important to monitor to determine if the mechanisms of action are addressed. Fidelity measures also include elements that may be altered through superficial adaptations. For example, fidelity tools for Problem Management Plus (PM+) were adapted in Nepal to capture the elements in each session that were adapted for the culture and context (Sangraula et al., 2021; Sangraula et al., 2020). Therefore, the cultural adaptation should include adapting the treatment

fidelity measure and capturing those components which are expected to be different from the original design.

References

Abi Ramia, J., Harper Shehadeh, M., Kheir, W., Zoghbi, E., Watts, S., Heim, E., & El Chammay, R. (2018). Community cognitive interviewing to inform local adaptations of an e-mental health intervention in Lebanon. *Global Mental Health, 5*, e39. https://doi.org/10.1017/gmh.2018.29

American Psychiatric Association. (2013). *Diagnostic and statistical manual of mental disorders (5th ed.)*. American Psychiatric Publishing.

Bernal, G., Bonilla, J., & Bellido, C. (1995). Ecological validity and cultural sensitivity for outcome research: issues for the cultural adaptation and development of psychosocial treatments with Hispanics. *Journal of Abnormal Child Psychology, 23*, 67–82.

Bernal, G., & Sáez-Santiago, E. (2006). Culturally centered psychosocial interventions. *Journal of Community Psychology, 34*(2), 121-132. https://doi.org/10.1002/jcop.20096

Boivin, A., Richards, T., Forsythe, L., Grégoire, A., L'Espérance, A., Abelson, J., & Carman, K. L. (2018). Evaluating patient and public involvement in research. *BMJ, 363*, k5147. https://doi.org/10.1136/bmj.k5147

Böttche, M., Kampisiou, C., Stammel, N., El-Haj-Mohamad, R., Heeke, C., Burchert, S., Heim, E., Wagner, B., Renneberg, B., Böttcher, J., Glaesmer, H., Gouzoulis-Mayfrank, E., Zielasek, J., Konnopka, A., Murray, L. K., & Knaevelsrud, C. (2021). From formative research to adaptation of an evidence-based cognitive-behavioural intervention: Decision-making processes for face-to-face and internet-based cultural adaptation for Arabic-speaking refugees in Germany. *Clinical Psychology in Europe, 3*(Special Issue), Article e4623. https://doi.org/https://doi.org/10.32872/cpe.4623

Burchert, S., Alkneme, M. S., Bird, M., Carswell, K., Cuijpers, P., Hansen, P., Heim, M., Harper Shehadeh, M., Sijbrandij, M., Van't Hof, E., & Knaevelsrud, C. (2019). User-centered app adaptation of a low-intensity e-mental health intervention for Syrian refugees. *Frontiers in Psychiatry, 9*, 663.

Carswell, K., Harper-Shehadeh, M., Watts, S., van't Hof, E., Abi Ramia, J., Heim, E., Wenger, A., & van Ommeren, M. (2018). Step-by-Step: a new WHO digital mental health intervention for depression. *mHealth, 4*(8). http://mhealth.amegroups.com/article/view/20772

Chu, J., & Leino, A. (2017). Advancement in the maturing science of cultural adaptations of evidence-based interventions. *Journal of Consulting and Clinical Psychology, 85*(1), 45-57. https://doi.org/10.1037/ccp0000145

Collins, S. E., Clifasefi, S. L., Stanton, J., The Leap Advisory, B., Straits, K. J. E., Gil-Kashiwabara, E., Rodriguez Espinosa, P., Nicasio, A. V., Andrasik, M. P., Hawes, S. M., Miller, K. A., Nelson, L. A., Orfaly, V. E., Duran, B. M., & Wallerstein, N. (2018, Oct). Community-based participatory research (CBPR): Towards equitable involvement of community in psychology research. *American Psychologist, 73*(7), 884-898. https://doi.org/10.1037/amp0000167

Cork, C., Kaiser, B. N., & White, R. G. (2019). The integration of idioms of distress into mental health assessments and interventions: a systematic review. *Global Mental Health, 6*, e7, Article e7. https://doi.org/10.1017/gmh.2019.5

Cuijpers, P., Cristea, I. A., Karyotaki, E., Reijnders, M., & Hollon, S. D. (2017). Component studies of psychological treatments of adult depression: A systematic review and meta-analysis. *Psychotherapy Research*, 1-15. https://doi.org/10.1080/10503307.2017.1395922

Cuijpers, P., Reijnders, M., & Huibers, M. J. H. (2019, May 7). The role of common ractors in psychotherapy outcomes. *Annual Review of Clinical Psychology, 15*, 207-231. https://doi.org/10.1146/annurev-clinpsy-050718-095424

Dawson, K. S., Bryant, R. A., Harper, M., Kuowei Tay, A., Rahman, A., Schafer, A., & van Ommeren, M. (2015). Problem Management Plus (PM+): a WHO transdiagnostic psychological intervention for common mental health problems. *World Psychiatry, 14*(3), 354-357. https://doi.org/10.1002/wps.20255

Dickerson, D., Baldwin, J. A., Belcourt, A., Belone, L., Gittelsohn, J., Keawe'aimoku Kaholokula, J., Lowe, J., Patten, C. A., & Wallerstein, N. (2020, Jan). Encompassing cultural contexts within scientific research methodologies in the development of health promotion interventions. *Prevention Science, 21*(Suppl 1), 33-42. https://doi.org/10.1007/s11121-018-0926-1

Ferster, C. B. (1973). A functional analysis of depression. *American Psychologist, 28*(10), 857-870. https://doi.org/10.1037/h0035605

Garabiles, M. R., Harper Shehadeh, M., & Hall, B. J. (2019). Cultural adaptation of a scalable World Health Organization e-mental health program for overseas Filipino workers [Original Paper]. *JMIR Formative Research, 3*(1), e11600. https://doi.org/10.2196/11600

Gearing, R. E., Schwalbe, C. S., MacKenzie, M. J., Brewer, K. B., Ibrahim, R. W., Olimat, H. S., Al-Makhamreh, S. S., Mian, I., & Al-Krenawi, A. (2012, 2013/11/01). Adaptation and translation of mental health interventions in Middle Eastern Arab countries: A systematic review of barriers to and strategies for effective treatment implementation. *International Journal of Social Psychiatry, 59*(7), 671-681. https://doi.org/10.1177/0020764012452349

Greene, M. C., Jordans, M. J. D., Kohrt, B. A., Ventevogel, P., Kirmayer, L. J., Hassan, G., Chiumento, A., van Ommeren, M., & Tol, W. A. (2017, November 15). Addressing culture and context in

humanitarian response: preparing desk reviews to inform mental health and psychosocial support [journal article]. *Conflict and Health, 11*(1), 21. https://doi.org/10.1186/s13031-017-0123-z

Groleau, D., Young, A., & Kirmayer, L. J. (2006, Dec). The McGill Illness Narrative Interview (MINI): An interview schedule to elicit meanings and modes of reasoning related to illness experience. *Transcultural Psychiatry, 43*(4), 671-691. https://doi.org/10.1177/1363461506070796

Haroz, E. E., Ritchey, M., Bass, J. K., Kohrt, B. A., Augustinavicius, J., Michalopoulos, L., Burkey, M. D., & Bolton, P. (2017, 2017/06/01/). How is depression experienced around the world? A systematic review of qualitative literature. *Social Science & Medicine, 183*, 151-162. https://doi.org/10.1016/j.socscimed.2016.12.030

Heim, E., Burchert, S., Shala, M., Kaufmann, M., Cerga-Pashoja, A., Morina, N., Schaub, M. P., Knaevelsrud, C., & Maercker, A. (2020). Effect of cultural adaptation of a smartphone-based self-help programme on its acceptability and efficacy: study protocol for a randomized controlled trial. *PsychArchives*. https://doi.org/10.23668/psycharchives.3152

Heim, E., & Knaevelsrud, C. (2021). Standardised research methods and documentation in cultural adaptation: the need, the potential and future steps. *Clinical Psychology in Europe, 3*(Special Issue), Article e5513. https://doi.org/https://doi.org/10.32872/cpe.5513

Heim, E., Mewes, R., Abi Ramia, J., Glaesmer, H., Hall, B., Harper Shehadeh, M., Ünlü, B., Kananian, S., Kohrt, B. A., Lechner-Meichsner, F., Lotzin, A., Moro, M. R., Radjack, R., Salamanca-Sanabria, A., Singla, D. R., Starck, A., Sturm, G., Tol, W., Weise, C., & Knaevelsrud, C. (2021). Supplementary materials to "Reporting Cultural Adaptation in Psychological Trials – The RECAPT criteria“ [RECAPT Template]. https://doi.org/https://doi.org/10.23668/psycharchives.5192

Hinton, D. E., & Otto, M. W. (2006, Nov 1). Symptom presentation and symptom meaning among traumatized Cambodian refugees: relevance to a somatically focused cognitive-behavior therapy. *Cognitive and Behavioural Practice 13*(4), 249-260. https://doi.org/10.1016/j.cbpra.2006.04.006

Hinton, D. E., Seponski, D. M., Khann, S., Armes, S. E., Lahar, C. J., Kao, S., & Schunert, T. (2019, 2019/08/01). Culturally sensitive assessment of anxious-depressive distress in the Cambodian population: Avoiding category truncation. *Transcultural Psychiatry, 56*(4), 643-666. https://doi.org/10.1177/1363461519851609

Kagawa Singer, M., Dressler, W., George, S., Elwood, W., & Panel, T. (2015). *The cultural framework for health: An integrative approach for research and program design and evaluation*. National Institutes of Health.

Kananian, S., Starck, A., & Stangier, U. (2021). Cultural adaptation of CBT for Afghan refugees in Europe: A retrospective evaluation. *Clinical Psychology in Europe, 3*(Special Issue), Article e5271. https://doi.org/https://doi.org/10.32872/cpe.5271

Keys, H. M., Kaiser, B. N., Kohrt, B. A., Khoury, N. M., & Brewster, A. R. (2012, Aug). Idioms of distress, ethnopsychology, and the clinical encounter in Haiti's Central Plateau. *Social Science & Medicine, 75*(3), 555-564. https://doi.org/10.1016/j.socscimed.2012.03.040

Kohrt, B. A., & Hruschka, D. J. (2010). Nepali concepts of psychological trauma: The role of idioms of distress, ethnopsychology, and ethnophysiology in alleviating suffering and preventing stigma. *Culture, Medicine and Psychiatry, 34*(2), 10.1007/s11013-11010-19170-11012. https://doi.org/10.1007/s11013-010-9170-2

Kohrt, B. A., Maharjan, S. M., Timsina, D., & Griffith, J. L. (2012, 2012/05/01). Applying Nepali ethnopsychology to psychotherapy for the treatment of mental illness and prevention of suicide among Bhutanese refugees. *Annals of Anthropological Practice, 36*(1), 88-112. https://doi.org/10.1111/j.2153-9588.2012.01094.x

Kohrt, B. A., Rasmussen, A., Kaiser, B. N., Haroz, E. E., Maharjan, S. M., Mutamba, B. B., de Jong, J. T., & Hinton, D. E. (2014, Apr). Cultural concepts of distress and psychiatric disorders: Literature review and research recommendations for global mental health epidemiology. *International Journal of Epidemiology, 43*(2), 365-406. https://doi.org/10.1093/ije/dyt227

Lemmens, L., Muller, V., Arntz, A., & Huibers, M. J. H. (2016, Dec). Mechanisms of change in psychotherapy for depression: An empirical update and evaluation of research aimed at identifying psychological mediators. *Clinical Psychology Review, 50*, 95-107. https://doi.org/10.1016/j.cpr.2016.09.004

Lewinsohn, P. M. (1974). A behavioral approach to depression. In R. J. Freidman & M. Katz (Eds.), *The psychology of depression: contemporary theory and research* (pp. 157-178). Wiley.

Lloyd, K. R., Jacob, K. S., Patel, V., St Louis, L., Bhugra, D., & Mann, A. H. (1998, Sep). The development of the Short Explanatory Model Interview (SEMI) and its use among primary-care attenders with common mental disorders. *Psychological Medicine, 28*(5), 1231-1237.

Lotzin, A., Lindert, J., Koch, T., Liedl, A., & Schäfer, I. (2021). STARC-SUD – Adaptation of a transdiagnostic intervention for refugees with substance use disorders. *Clinical Psychology in Europe, 3*(Special Issue), Article e5329. https://doi.org/https://doi.org/10.32872/cpe.5329

Malterud, K. (2001, 2001/08/11/). Qualitative research: standards, challenges, and guidelines. *The Lancet, 358*(9280), 483-488. https://doi.org/https://doi.org/10.1016/S0140-6736(01)05627-6

Mummah, S. A., Robinson, T. N., King, A. C., Gardner, C. D., & Sutton, S. (2016). IDEAS (Integrate, Design, Assess, and Share): A Framework and Toolkit of Strategies for the Development of

More Effective Digital Interventions to Change Health Behavior [Viewpoint]. *Journal of Medical Internet Research, 18*(12), e317. https://doi.org/10.2196/jmir.5927

Napier, A. D., Ancarno, C., Butler, B., Calabrese, J., Chater, A., Chatterjee, H., Guesnet, F., Horne, R., Jacyna, S., Jadhav, S., Macdonald, A., Neuendorf, U., Parkhurst, A., Reynolds, R., Scambler, G., Shamdasani, S., Smith, S. Z., Stougaard-Nielsen, J., Thomson, L., Tyler, N., Volkmann, A.-M., Walker, T., Watson, J., de C Williams, A. C., Willott, C., Wilson, J., & Woolf, K. (2014). Culture and health. *The Lancet, 384*(9954), 1607-1639. https://doi.org/10.1016/S0140-6736(14)61603-2

Perera, C., Salamanca-Sanabria, A., Caballero-Bernal, J., Feldman, L., Hansen, M., Bird, M., Hansen, P., Dinesen, C., Wiedemann, N., & Vallières, F. (2020, 2020/07/14). No implementation without cultural adaptation: a process for culturally adapting low-intensity psychological interventions in humanitarian settings. *Conflict and Health, 14*(1), 46. https://doi.org/10.1186/s13031-020-00290-0

Price, A., Schroter, S., Snow, R., Hicks, M., Harmston, R., Staniszewska, S., Parker, S., & Richards, T. (2018). Frequency of reporting on patient and public involvement (PPI) in research studies published in a general medical journal: a descriptive study. *BMJ open, 8*(3), e020452. https://doi.org/10.1136/bmjopen-2017-020452

Reich, H., Zürn, D., & Mewes, R. (in press). Engaging Turkish immigrants in psychotherapy: development and proof-of-concept study of a culture-tailored, web-based intervention. *Clinical Psychology in Europe*. https://doi.org/https://www.psycharchives.org/handle/20.500.12034/4585

Resnicow, K., Baranowski, T., Ahluwalia, J. S., & Braithwaite, R. L. (1999, Winter). Cultural sensitivity in public health: defined and demystified. *Ethnicity & Disease, 9*(1), 10-21.

Rüdell, K., Bhui, K., & Priebe, S. (2009, March 1, 2009). Concept, development and application of a new mixed method assessment of cultural variations in illness perceptions: Barts explanatory model inventory. *Journal of Health Psychology, 14*(2), 336-347. https://doi.org/10.1177/1359105308100218

Salamanca-Sanabria, A., Richards, D., & Timulak, L. (2019). Adapting an internet-delivered intervention for depression for a Colombian college student population: An illustration of an integrative empirical approach. *Internet Interventions, 15*, 76-86. https://doi.org/10.1016/j.invent.2018.11.005

Sangraula, M., Kohrt, B. A., Ghimire, R., Shrestha, P., Luitel, N. P., van't Hof, E., Dawson, K., & Jordans, M. J. D. (2021). Development of the mental health cultural adaptation and contextualization for implementation (mhCACI) procedure: a systematic framework to prepare evidence-based

psychological interventions for scaling. *Global Mental Health, 8*, e6, Article e6. https://doi.org/10.1017/gmh.2021.5

Sangraula, M., Turner, E. L., Luitel, N. P., van 't Hof, E., Shrestha, P., Ghimire, R., Bryant, R., Marahatta, K., van Ommeren, M., Kohrt, B. A., & Jordans, M. J. D. (2020). Feasibility of Group Problem Management Plus (PM+) to improve mental health and functioning of adults in earthquake-affected communities in Nepal. *Epidemiology and Psychiatric Sciences, 29*, e130-e130. https://doi.org/10.1017/S2045796020000414

Shala, M., Morina, N., Burchert, S., Cerga-Pashoja, A., Knaevelsrud, C., Maercker, A., & Heim, E. (2020, 2020/09/01/). Cultural adaptation of Hap-pas-Hapi, an internet and mobile-based intervention for the treatment of psychological distress among Albanian migrants in Switzerland and Germany. *Internet Interventions, 21*, 100339. https://doi.org/10.1016/j.invent.2020.100339

Shala, M., Morina, N., Salis Gross, C., Maercker, A., & Heim, E. (2020, 2019/06/17). A point in the heart: concepts of emotional distress among Albanian-speaking immigrants in Switzerland. *Culture, Medicine, and Psychiatry, 40*, 1–34. https://doi.org/10.1007/s11013-019-09638-5

van Ommeren, M., Sharma, B., Thapa, S., Makaju, R., Prasain, D., Bhattarai, R., & de Jong, J. (1999, 1999/09/01). Preparing instruments for transcultural research: use of the translation monitoring form with Nepali-speaking Bhutanese refugees. *Transcultural Psychiatry, 36*(3), 285-301. https://doi.org/10.1177/136346159903600304

Veale, D. (2008). Behavioural activation for depression. *Advances in Psychiatric Treatment, 14*(1), 29-36. https://doi.org/10.1192/apt.bp.107.004051

Walters, K. L., Johnson-Jennings, M., Stroud, S., Rasmus, S., Charles, B., John, S., Allen, J., Kaholokula, J. K., Look, M. A., de Silva, M., Lowe, J., Baldwin, J. A., Lawrence, G., Brooks, J., Noonan, C. W., Belcourt, A., Quintana, E., Semmens, E. O., & Boulafentis, J. (2020, Jan). Growing from our roots: strategies for developing culturally grounded health promotion interventions in American Indian, Alaska Native, and Native Hawaiian communities. *Prevention Science, 21*(Suppl 1), 54-64. https://doi.org/10.1007/s11121-018-0952-z

Wampold, B. (2007, Nov). Psychotherapy: the humanistic (and effective) treatment. *American Psychologist, 62*(8), 855-873. https://doi.org/10.1037/0003-066x.62.8.857

Wampold, B. (2015). How important are the common factors in psychotherapy? An update. *World Psychiatry 14*(3), 270-277. https://doi.org/10.1002/wps.20238

White, G. M. (1992). Ethnopsychology. In C. Lutz, G. M. White, & T. Schwartz (Eds.), *New directions in psychological anthropology* (pp. 21-46). Cambridge University Press.

Willis, G. B. (2004). *Cognitive interviewing: A tool for improving questionnaire design*. Sage Publications.

Wingood, G. M., & DiClemente, R. J. (2008). The ADAPT-ITT model: A novel method of adapting evidence-based HIV interventions. *JAIDS Journal of Acquired Immune Deficiency Syndromes, 47*. https://journals.lww.com/jaids/Fulltext/2008/03011/The_ADAPT_ITT_Model__A_Novel_Method_of_Adapting.8.aspx

Appendix B: Expert Survey

Questionnaire

1 Welcome

Dear colleague

Many thanks for participating in our expert survey. We are a group of researchers "Task force for cultural adaptions of mental health interventions for refugees", funded by the German Federal Ministry of Education and Research (BMBF).

We are in the process of developing **standard reporting criteria for cultural adaptation in psychological trials** and would be delighted to receive your contribution.

The reporting criteria will be published as part of a **special issue** on cultural adaptation in psychological trials. Experts who participate in the survey will be invited as **co-authors** for this publication.

Please do not hesitate to contact me if you have any question or comment: e.heim@psychologie.uzh.ch

Kind regards

Eva Heim

Please enter here your personal code, which you have received by mail

2 REporting Cultural Adaptation in Psychological Trials (RECAPT): List of criteria

Introduction

Evidence indicates that the cultural adaptation of psychological interventions for the treatment of common mental disorders increases their acceptability and efficacy (Hall et al., 2016; Harper Shehadeh et al., 2016). There is a large variety of target populations, psychological interventions and settings where cultural adaptation may occur, from low-intensity interventions in humanitarian settings (Perera et al., 2020) to higher-intensity interventions through the internet (Knaevelsrud et al., 2015) or face-to-face (Hinton et al., 2012), to mention only a few. Most studies use a top-down approach, in which existing psychological interventions developed for one cultural group are adapted for another one. Few studies use a bottom-up approach to develop new interventions based on culturally specific symptoms or syndromes (Hall et al., 2016; Hwang, 2006).

So far, there are no standard criteria for the documentation of bottom-up and top-down cultural adaptations in clinical trials testing psychological interventions (in short: psychological trials). In the following, we provide a list of such reporting criteria. In order to increase the quality, validity and comparability of empirical evidence on psychological interventions for diverse ethnic and cultural groups, we recommend to report on the following criteria in psychological trials, regardless of whether they were implemented or not. These reporting criteria can also be used as a guideline for planning the process of cultural adaptation of an existing intervention (top-down), or the consideration of cultural aspects in the development of new interventions (bottom-up) to be tested in psychological trials.

Several frameworks for cultural adaptation of evidence-based interventions exist (e.g., Castro et al., 2010; Perera et al., 2020), all of which have been developed mainly for clinical practice (as opposed to research). These frameworks have in common that they use stage models which include assessment, selection of intervention (components), adaptation, piloting, and implementation. A prominent framework was developed by the Applied Mental Health Research (AMHR) Group at Johns Hopkins University: The Development, Implementation, Monitoring, and Evaluation (DIME) manual to support programs among trauma-affected populations (AMHR, 2013). The DIME manual aims to (1) identify and measure the impact and prevalence of mental health and psychosocial problems in the populations they seek to serve; (2) develop or adapt appropriate interventions to address these problems; and

(3) measure the impact of these interventions.

Resnicow et al. (1999) differentiate between surface and deep structure adaptations of evidence-based interventions in health promotion and prevention in general. Surface structure adaptations refer to matching materials (e.g., illustrations, language), as well as channels and settings for treatment delivery to observable characteristics of the target population. Thus, surface adaptations foster the *acceptance* of an intervention. By contrast, deep structure adaptations take into account how cultural, social, environmental, or historical factors influence health behaviours. Such adaptations are based on assumptions of how members of a particular cultural group perceive the cause, course, and treatment of a particular illness. Deep structure adaptations convey salience of an intervention and determine its *efficacy* or impact.

In psychological interventions, efficacy is achieved through the application of specific and unspecific elements, and therapeutic techniques (Singla et al., 2017). Elements are therapeutic activities or strategies, whereas techniques are skills that the therapist implements during a session. Specific elements are grounded in specific psychological mechanisms (i.e., behavioural, cognitive, emotional, and interpersonal elements). Unspecific elements are routed in the “common factors” of psychological interventions, such as therapeutic alliance, positive expectations, and providing a convincing treatment rationale (Cuijpers et al., 2019; Wampold, 2007).

Based on these different frameworks, the list of reporting criteria for bottom-up and top-down cultural adaptation in psychological trials is structured along the following categories: A) Set-up; B) Formative research; C) Intervention; D) Measuring outcomes. The last category, measuring outcomes, is kept short, as this is addressed in specific literature. However, since measuring outcomes is an integral part of randomised controlled trials, we decided to include it as part of the reporting criteria.

If possible, we recommend to publish a separate paper on formative research and cultural adaptation alongside the regular papers of a psychological trial (i.e., protocol and results paper), as it has been done in several studies (e.g., Abi Ramia et al., 2018). If it is not possible to publish a separate paper on the formative research and cultural adaptation, it is still recommendable to report on the most important aspects in the protocol or results paper.

Do you have any comments on the introduction?

3 Instruction

Instruction

In the following, we will show you the 11 reporting criteria suggested by the task force. An overview of the 11 criteria is provided on the next page.

The criteria are divided in four sections. Each section starts with a short introduction. You are invited to provide comments on the introduction text.

For each criterion, we will ask you to rate how much you agree with the criterion itself and with the description. In addition, you can provide feedback on the criterion in an open text field.

Thereafter, we will ask you to rate for each criterion a) the relevance; and b) the applicability.

Thank you very much for your valuable contribution.

4 REporting Cultural Adaptation in Psychological Trials (RECAPT): List o...

REporting Cultural Adaptation in Psychological Trials (RECAPT): List of criteria

A) Set-up
Criterion 1. Team and roles
Criterion 2. Establishing a documentation and monitoring system
Criterion 3. Documentation of adaptations during trials ("on the fly")

B) Formative research
Criterion 4. Methods
Criterion 5. Definition of the target population
Criterion 6. Target symptoms, syndromes, and needs

C) Intervention
Criterion 7. Specific elements
Criterion 8. Unspecific elements and therapeutic techniques
Criterion 9. Surface adaptations

D) Measuring outcomes
Criterion 10. Questionnaires
Criterion 11. Clinical interview

5 A) Set-up

A) Set-up

Cultural adaptation of psychological interventions is a complex process which most often includes several stages. Once a psychological trial is completed and results are about to be published, it may be difficult or impossible to reconstruct all the decisions that were made during the cultural adaptation process. For this reason, it is recommendable to continuously document this process, and to be explicit about the people involved in decision-making.

Do you have any comments on the text?

6 Criterion 1. Team and roles

Criterion 1. Team and roles

Several guidelines for qualitative research (e.g., Malterud, 2001; Tong et al., 2007) consistently recommend to provide information on the personal characteristics of the researchers involved in qualitative studies (e.g., occupation, gender, experience and training), as well as information about preconceptions, which represent previous experiences, prestudy beliefs, motivation and qualifications. As Malterud (2001) outlines, providing this

information contributes to the process of reflexivity and accounts for the view that knowledge is partial and situated. Providing information about the researchers' background and preconceptions may contribute to the reflection about what aspects in the encounter between researchers and study participants may have contributed to or shaped the results, and therefore reduce bias. In this sense, it is recommended to shortly describe the team that was involved in the cultural adaptation process, as well as their roles during the formative research phase and in the decision-making process. An example was provided on the cultural adaptation of an online self-help intervention for overseas Filipino workers in Macau (Garabiles et al., 2019).

Do you agree with this criterion?

- ○ Strongly agree
- ○ Somewhat agree
- ○ Somewhat disagree
- ○ Strongly disagree

7 Criterion 1. Team and roles

Criterion 1. Team and roles

Several guidelines for qualitative research (e.g., Malterud, 2001; Tong et al., 2007) consistently recommend to provide information on the personal characteristics of the researchers involved in qualitative studies (e.g., occupation, gender, experience and training), as well as information about preconceptions, which represent previous experiences, prestudy beliefs, motivation and qualifications. As Malterud (2001) outlines, providing this information contributes to the process of reflexivity and accounts for the view that knowledge is partial and situated. Providing information about the researchers' background and preconceptions may contribute to the reflection about what aspects in the encounter between researchers and study participants may have contributed to or shaped the results, and therefore reduce bias. In this sense, it is recommended to shortly describe the team that was involved in the cultural adaptation process, as well as their roles during the formative research phase and in the decision-making process. An example was provided on the cultural adaptation of an online self-help intervention for overseas Filipino workers in Macau (Garabiles et al., 2019).

Do you agree with the description?

- ○ Strongly agree
- ○ Somewhat agree
- ○ Somewhat disagree
- ○ Strongly disagree

8 Criterion 1. Team and roles

Criterion 1. Team and roles

Several guidelines for qualitative research (e.g., Malterud, 2001; Tong et al., 2007) consistently recommend to provide information on the personal characteristics of the researchers involved in qualitative studies (e.g., occupation, gender, experience and training), as well as information about preconceptions, which represent previous experiences, prestudy beliefs, motivation and qualifications. As Malterud (2001) outlines, providing this information contributes to the process of reflexivity and accounts for the view that knowledge is partial and situated. Providing information about the researchers' background and preconceptions may contribute to the reflection about what aspects in the encounter between researchers and study participants may have contributed to or shaped the results, and therefore reduce bias. In this sense, it is recommended to shortly describe the team that was involved in the cultural adaptation process, as well as their roles during the formative research phase and in the decision-making process. An example was provided on the cultural adaptation of an online self-help intervention for overseas Filipino workers in Macau (Garabiles et al., 2019).

Do you have any comments on the criterion and the text?

9 Criterion 1. Team and roles

Criterion 1. Team and roles

Please rate the relevance of this criterion

- ◯ Totally relevant
- ◯ Somewhat relevant
- ◯ Hardly relevant
- ◯ Not at all relevant

10 Criterion 1. Team and roles

Criterion 1. Team and roles

Please rate the applicability of this criterion

- ◯ Totally applicable
- ◯ Somewhat applicable

Hardly applicable

Not at all applicable

11 Criterion 2. Establishing a documentation and monitoring system

Criterion 2. Establishing a documentation and monitoring system

Shala, Morina, Burchert, et al. (2020) have developed a documentation and monitoring methodology for the cultural adaptation of an internet- and app-based psychological intervention. This methodology is based on a conceptual framework for cultural adaptation of psychological interventions by Heim and Kohrt (2019). The methodology was applied and further developed in the project "Task force for cultural adaptions of mental health interventions for refugees", funded by the German Federal Ministry of Education and Research (BMBF). In this project, the process of cultural adaptation was harmonised across six studies to test psychological interventions with refugee populations in Germany. A template for documenting cultural adaptations can be found in (Introduction paper of special issue). For reasons of transparency, we recommend to add the documentation and monitoring sheet as supplementary material to published papers.

When documenting the process of cultural adaptation, we recommend to provide information on the source of information for each decision, and on the strength of evidence to support decisions. In formative research, many suggestions for changing and adapting parts of the intervention may be made. Some of these suggestions may be "nice-to-have", or even controversial, especially if they are based on personal taste. On the other hand, there may be changes that are absolutely essential, because not doing them causes either harm (e.g., hurting feelings of subgroups) or makes participants leave the intervention. If particular aspects are mentioned by different key informants or consistently across different focus groups, this may be strong evidence for a necessary adaptation. By contrast, if a suggestion is based on one individual opinion, this is considered to be weak evidence. Based on the chosen methods of formative research, it may be recommendable to pre-define criteria for decision-making and the required strength of evidence for such decisions. Since most of the times not all adaptions can be made in the very beginning, the system should also have a section for documenting adaptions later on.

Do you agree with this criterion?

Strongly agree

Somewhat agree

Somewhat disagree

Strongly disagree

12 Criterion 2. Establishing a documentation and monitoring system

Criterion 2. Establishing a documentation and monitoring system

Shala, Morina, Burchert, et al. (2020) have developed a documentation and monitoring methodology for the cultural adaptation of an internet- and app-based psychological intervention. This methodology is based on a conceptual framework for cultural adaptation of psychological interventions by Heim and Kohrt (2019). The methodology was applied and further developed in the project "Task force for cultural adaptions of mental health

interventions for refugees”, funded by the German Federal Ministry of Education and Research (BMBF). In this project, the process of cultural adaptation was harmonised across six studies to test psychological interventions with refugee populations in Germany. A template for documenting cultural adaptations can be found in (Introduction paper of special issue). For reasons of transparency, we recommend to add the documentation and monitoring sheet as supplementary material to published papers.

When documenting the process of cultural adaptation, we recommend to provide information on the source of information for each decision, and on the strength of evidence to support decisions. In formative research, many suggestions for changing and adapting parts of the intervention may be made. Some of these suggestions may be “nice-to-have”, or even controversial, especially if they are based on personal taste. On the other hand, there may be changes that are absolutely essential, because not doing them causes either harm (e.g., hurting feelings of subgroups) or makes participants leave the intervention. If particular aspects are mentioned by different key informants or consistently across different focus groups, this may be strong evidence for a necessary adaptation. By contrast, if a suggestion is based on one individual opinion, this is considered to be weak evidence. Based on the chosen methods of formative research, it may be recommendable to pre-define criteria for decision-making and the required strength of evidence for such decisions. Since most of the times not all adaptions can be made in the very beginning, the system should also have a section for documenting adaptions later on.

Do you agree with the description?

- ○ Strongly agree
- ○ Somewhat agree
- ○ Somewhat disagree
- ○ Strongly disagree

13 Criterion 2. Establishing a documentation and monitoring system

Criterion 2. Establishing a documentation and monitoring system

Shala, Morina, Burchert, et al. (2020) have developed a documentation and monitoring methodology for the cultural adaptation of an internet- and app-based psychological intervention. This methodology is based on a conceptual framework for cultural adaptation of psychological interventions by Heim and Kohrt (2019). The methodology was applied and further developed in the project “Task force for cultural adaptions of mental health interventions for refugees”, funded by the German Federal Ministry of Education and Research (BMBF). In this project, the process of cultural adaptation was harmonised across six studies to test psychological interventions with refugee populations in Germany. A template for documenting cultural adaptations can be found in (Introduction paper of special issue). For reasons of transparency, we recommend to add the documentation and monitoring sheet as supplementary material to published papers.

When documenting the process of cultural adaptation, we recommend to provide information on the source of information for each decision, and on the strength of evidence to support decisions. In formative research, many suggestions for changing and adapting parts of the intervention may be made. Some of these suggestions may be “nice-to-have”, or even controversial, especially if they are based on personal taste. On the other hand, there may be changes that are absolutely essential, because not doing them causes either harm (e.g., hurting feelings of subgroups) or makes participants leave the intervention. If particular aspects are mentioned by different key informants or consistently across different focus groups, this may be strong evidence for a necessary adaptation.

By contrast, if a suggestion is based on one individual opinion, this is considered to be weak evidence. Based on the chosen methods of formative research, it may be recommendable to pre-define criteria for decision-making and the required strength of evidence for such decisions. Since most of the times not all adaptions can be made in the very beginning, the system should also have a section for documenting adaptions later on.

Do you have any comments on the criterion and the text?

14 Criterion 2. Establishing a documentation and monitoring system

Criterion 2. Establishing a documentation and monitoring system

Please rate the relevance of this criterion

- Totally relevant
- Somewhat relevant
- Hardly relevant
- Not at all relevant

15 Criterion 2. Establishing a documentation and monitoring system

Criterion 2. Establishing a documentation and monitoring system

Please rate the applicability of this criterion

- Totally applicable
- Somewhat applicable
- Hardly applicable
- Not at all applicable

16 Criterion 3. Documentation of adaptations during trials (“on the fly”)

Criterion 3. Documentation of adaptations during trials ("on the fly")

In their publication entitled “The Adaptome - Advancing the Science of Intervention Adaptation”, Chambers and Norton (2016) challenge the assumption of a linear, static process from intervention development (and adaptation) to pilot testing, randomised controlled trial, and implementation. In this linear view that is still prevailing in literature, deviances from manuals are considered to be problematic, as they may threaten treatment fidelity and thus, effectiveness of the intervention. In the Adaptome, Chambers and Norton (2016) aim to capture “positive deviance (e.g., where adaptation leads to better outcomes compared to the original trials) as well as circumstances in which program drift was deleterious to intervention effectiveness” (p. 127).

In most running trials, adaptations happen “on the fly”, and especially when working with diverse ethnic and cultural groups, for whom we have less empirical evidence on psychological interventions. It is thus recommendable to have a system in place for decision-making and documentation of such deviations. As an example, if a misunderstanding in psychoeducation is discovered, it might be necessary to adapt the wording and, if needed, provide standard translations of such psychoeducation to interpreters for the rest of the trial (citation: paper in special issue). In case of a multi-center trial, it is important to decide how relevant information on such on-the-fly adaptations should be reported back to principal investigators, and who is entitled to make decisions on such adaptations. In addition, using a standard documentation system (see above) will enhance transparency on adaptations that were made during trials. As Chambers et al. (2013) state: “By augmenting trial data with practice-based evidence, we can understand much more about what works for whom” (p. 6).

Due to the current procedures with ethical protocols and trial registration, in which all relevant changes have to be approved by ethical review committees in amendments to protocols, researchers might be hesitant to document changes in procedures or interventions in running trials. And one may argue that ideally, such difficulties are discovered in pilot trials that are done exactly for this purpose. However, it is still possible that important information is revealed in the course of running trials, and documentation and transparency with regard to such “on-the-fly” adaptations may be relevant for a better understanding of trial results and implementation. We recommend using a documentation and monitoring sheet (see above) and add this information as supplementary material to the published paper on the main results of the trial, or the separate paper on cultural adaptation.

Do you agree with this criterion?

- Strongly agree
- Somewhat agree
- Somewhat disagree
- Strongly disagree

17 Criterion 3. Documentation of adaptations during trials (“on the fly”)

Criterion 3. Documentation of adaptations during trials ("on the fly")

In their publication entitled “The Adaptome - Advancing the Science of Intervention Adaptation”, Chambers and Norton (2016) challenge the assumption of a linear, static process from intervention development (and adaptation) to pilot testing, randomised controlled trial, and implementation. In this linear view that is still prevailing in literature, deviances from manuals are considered to be problematic, as they may threaten treatment fidelity and thus, effectiveness of the intervention. In the Adaptome, Chambers and Norton (2016) aim to capture “positive deviance (e.g., where adaptation leads to better outcomes compared to the original trials) as well as circumstances in which program drift was deleterious to intervention effectiveness” (p. 127).

In most running trials, adaptations happen “on the fly”, and especially when working with diverse ethnic and cultural groups, for whom we have less empirical evidence on psychological interventions. It is thus recommendable to have a system in place for decision-making and documentation of such deviations. As an example, if a misunderstanding in psychoeducation is discovered, it might be necessary to adapt the wording and, if needed, provide standard translations of such psychoeducation to interpreters for the rest of the trial (citation: paper in special issue). In case of a multi-center trial, it is important to decide how relevant information on such on-the-fly adaptations should be reported back to principal investigators, and who is entitled to make decisions on such adaptations. In addition, using a standard documentation system (see above) will enhance transparency on adaptations that were made during trials. As Chambers et al. (2013) state: “By augmenting trial data with practice-based evidence, we can understand much more about what works for whom” (p. 6).

Due to the current procedures with ethical protocols and trial registration, in which all relevant changes have to be approved by ethical review committees in amendments to protocols, researchers might be hesitant to document changes in procedures or interventions in running trials. And one may argue that ideally, such difficulties are discovered in pilot trials that are done exactly for this purpose. However, it is still possible that important information is revealed in the course of running trials, and documentation and transparency with regard to such “on-the-fly” adaptations may be relevant for a better understanding of trial results and implementation. We recommend using a documentation and monitoring sheet (see above) and add this information as supplementary material to the published paper on the main results of the trial, or the separate paper on cultural adaptation.

Do you agree with the description?

- ○ Strongly agree
- ○ Somewhat agree
- ○ Somewhat disagree
- ○ Strongly disagree

18 Criterion 3. Documentation of adaptations during trials (“on the fly”)

Criterion 3. Documentation of adaptations during trials ("on the fly")

In their publication entitled “The Adaptome - Advancing the Science of Intervention Adaptation”, Chambers and Norton (2016) challenge the assumption of a linear, static process from intervention development (and adaptation) to pilot testing, randomised controlled trial, and implementation. In this linear view that is still prevailing in literature, deviances from manuals are considered to be problematic, as they may threaten treatment fidelity and thus, effectiveness of the intervention. In the Adaptome, Chambers and Norton (2016) aim to capture “positive deviance (e.g., where adaptation leads to better outcomes compared to the original trials) as well as circumstances in which program drift was deleterious to intervention effectiveness” (p. 127).

In most running trials, adaptations happen “on the fly”, and especially when working with diverse ethnic and cultural groups, for whom we have less empirical evidence on psychological interventions. It is thus recommendable to have a system in place for decision-making and documentation of such deviations. As an example, if a misunderstanding in psychoeducation is discovered, it might be necessary to adapt the wording and, if needed, provide standard translations of such psychoeducation to interpreters for the rest of the trial (citation: paper in special issue). In case of a multi-center trial, it is important to decide how relevant information on such on-the-fly adaptations should be reported back to principal investigators, and who is entitled to make decisions on

such adaptations. In addition, using a standard documentation system (see above) will enhance transparency on adaptations that were made during trials. As Chambers et al. (2013) state: “By augmenting trial data with practice-based evidence, we can understand much more about what works for whom” (p. 6).

Due to the current procedures with ethical protocols and trial registration, in which all relevant changes have to be approved by ethical review committees in amendments to protocols, researchers might be hesitant to document changes in procedures or interventions in running trials. And one may argue that ideally, such difficulties are discovered in pilot trials that are done exactly for this purpose. However, it is still possible that important information is revealed in the course of running trials, and documentation and transparency with regard to such “on-the-fly” adaptations may be relevant for a better understanding of trial results and implementation. We recommend using a documentation and monitoring sheet (see above) and add this information as supplementary material to the published paper on the main results of the trial, or the separate paper on cultural adaptation.

Do you have any comments on the criterion and the text?

19 Criterion 3. Documentation of adaptations during trials (“on the fly”)

Criterion 3. Documentation of adaptations during trials (“on the fly”)

Please rate the relevance of this criterion

- Totally relevant
- Somewhat relevant
- Hardly relevant
- Not at all relevant

20 Criterion 3. Documentation of adaptations during trials (“on the fly”)

Criterion 3. Documentation of adaptations during trials (“on the fly”)

Please rate the applicability of this criterion

- Totally applicable
- Somewhat applicable
- Hardly applicable

Not at all applicable

21 B) Formative research

B) Formative research

Formative research includes the iterative process of gathering relevant information before starting a trial. The process of formative research is ideally reported in a consistent and transparent manner, to ensure replicability and valid interpretation of results. Our suggested reporting criteria include the *methods* of formative research on the one hand, and the *results* of this process on the other hand.

Do you have any comments on the text?

22 Criterion 4. Methods

Criterion 4. Methods

Formative research is an iterative process using multiple qualitative and quantitative methods. Results of this process should highlight the description of the target population's main characteristics, their most salient symptoms or syndromes and needs, and the feedback gathered on the intervention during the process of cultural adaptation. Although there is no "standard procedure" for top-down or bottom-up cultural adaptation, we suggest to report on these different stages of formative research.

Formative research normally starts with literature review. In their framework for the cultural adaptation of low-intensity psychological interventions in humanitarian settings, Perera et al. (2020) suggest performing a rapid desk review (Greene et al., 2017). In humanitarian situations, where a response needs to be provided immediately, it makes sense to speed up this process. By contrast, when testing new interventions in psychological trials, the process of literature review may take more time. Existing literature (including grey literature) on symptoms, syndromes and needs in the target population is needed as a starting point for bottom-up and top-down adaptation. In addition, it is relevant to identify literature on the cultural adaptation of psychological interventions for specific target groups, e.g. as done for Arab countries (Gearing et al., 2012).

Thereafter, qualitative and quantitative information on the target population (i.e., main characteristics, symptoms, syndromes, needs) is gathered where no or insufficient evidence is available. Mixed-methods approaches may be used to gather the most relevant information (Shala, Morina, Burchert, et al., 2020). Quantitative methods include symptoms scales, surveys, or other questionnaires used to describe the target population. Qualitative methods include in-depth interviews with key informants, focus groups, free-list interviews, pile sorting, among others (Cork et al., 2019; Keys et al., 2012). A multistakeholder-perspective is recommended, including clinicians, community members, potential users of the intervention, and other relevant groups.

We recommend using the consolidated criteria for reporting qualitative research (COREQ, Tong et al., 2007), a 32-items checklist for explicit and comprehensive reporting of qualitative studies. It includes participant selection (i.e.,

selection, method of approach, sample size, reasons for refusing); the setting for data collection (e.g., home, clinic); the method of data collection (i.e., interview guide, recording, duration), and analysis methods (i.e., how themes were derived from the data).

Once data on the target population is gathered and compiled, interventions are adapted in a bottom-up or top-down approach. And iterative process of adaptation, validation and piloting is recommended (e.g., Shala, Morina, Burchert, et al., 2020). Studies on cultural adaptation of psychological interventions have used focus groups and key informant interviews (Abi Ramia et al., 2018; Garabiles et al., 2019). In addition, "cognitive interviewing" (Willis, 2004) has been used frequently in literature, in which intervention materials are read through and participants "think aloud". Wingood and DiClemente (2008) suggest identifying "topical experts" who can assist in adaptation. And Salamanca-Sanabria et al. (2019) developed an instrument, the Cultural Relevance Questionnaire (CRQ) to support and document the cultural adaptation of psychological interventions.

Do you agree with this criterion?

- ○ Strongly agree
- ○ Somewhat agree
- ○ Somewhat disagree
- ○ Strongly disagree

23 Criterion 4. Methods

Criterion 4. Methods

Formative research is an iterative process using multiple qualitative and quantitative methods. Results of this process should highlight the description of the target population's main characteristics, their most salient symptoms or syndromes and needs, and the feedback gathered on the intervention during the process of cultural adaptation. Although there is no "standard procedure" for top-down or bottom-up cultural adaptation, we suggest to report on these different stages of formative research.

Formative research normally starts with literature review. In their framework for the cultural adaptation of low-intensity psychological interventions in humanitarian settings, Perera et al. (2020) suggest performing a rapid desk review (Greene et al., 2017). In humanitarian situations, where a response needs to be provided immediately, it makes sense to speed up this process. By contrast, when testing new interventions in psychological trials, the process of literature review may take more time. Existing literature (including grey literature) on symptoms, syndromes and needs in the target population is needed as a starting point for bottom-up and top-down adaptation. In addition, it is relevant to identify literature on the cultural adaptation of psychological interventions for specific target groups, e.g. as done for Arab countries (Gearing et al., 2012).

Thereafter, qualitative and quantitative information on the target population (i.e., main characteristics, symptoms, syndromes, needs) is gathered where no or insufficient evidence is available. Mixed-methods approaches may be used to gather the most relevant information (Shala, Morina, Burchert, et al., 2020). Quantitative methods include symptoms scales, surveys, or other questionnaires used to describe the target population. Qualitative methods include in-depth interviews with key informants, focus groups, free-list interviews, pile sorting, among others (Cork et al., 2019; Keys et al., 2012). A multistakeholder-perspective is recommended, including clinicians, community members, potential users of the intervention, and other relevant groups.

We recommend using the consolidated criteria for reporting qualitative research (COREQ, Tong et al., 2007), a 32-items checklist for explicit and comprehensive reporting of qualitative studies. It includes participant selection (i.e., selection, method of approach, sample size, reasons for refusing); the setting for data collection (e.g., home, clinic); the method of data collection (i.e., interview guide, recording, duration), and analysis methods (i.e., how themes were derived from the data).

Once data on the target population is gathered and compiled, interventions are adapted in a bottom-up or top-down approach. And iterative process of adaptation, validation and piloting is recommended (e.g., Shala, Morina, Burchert, et al., 2020). Studies on cultural adaptation of psychological interventions have used focus groups and key informant interviews (Abi Ramia et al., 2018; Garabiles et al., 2019). In addition, "cognitive interviewing" (Willis, 2004) has been used frequently in literature, in which intervention materials are read through and participants "think aloud". Wingood and DiClemente (2008) suggest identifying "topical experts" who can assist in adaptation. And Salamanca-Sanabria et al. (2019) developed an instrument, the Cultural Relevance Questionnaire (CRQ) to support and document the cultural adaptation of psychological interventions.

Do you agree with the description?

- ○ Strongly agree
- ○ Somewhat agree
- ○ Somewhat disagree
- ○ Strongly disagree

24 Criterion 4. Methods

Criterion 4. Methods

Formative research is an iterative process using multiple qualitative and quantitative methods. Results of this process should highlight the description of the target population's main characteristics, their most salient symptoms or syndromes and needs, and the feedback gathered on the intervention during the process of cultural adaptation. Although there is no "standard procedure" for top-down or bottom-up cultural adaptation, we suggest to report on these different stages of formative research.

Formative research normally starts with literature review. In their framework for the cultural adaptation of low-intensity psychological interventions in humanitarian settings, Perera et al. (2020) suggest performing a rapid desk review (Greene et al., 2017). In humanitarian situations, where a response needs to be provided immediately, it makes sense to speed up this process. By contrast, when testing new interventions in psychological trials, the process of literature review may take more time. Existing literature (including grey literature) on symptoms, syndromes and needs in the target population is needed as a starting point for bottom-up and top-down adaptation. In addition, it is relevant to identify literature on the cultural adaptation of psychological interventions for specific target groups, e.g. as done for Arab countries (Gearing et al., 2012).

Thereafter, qualitative and quantitative information on the target population (i.e., main characteristics, symptoms, syndromes, needs) is gathered where no or insufficient evidence is available. Mixed-methods approaches may be used to gather the most relevant information (Shala, Morina, Burchert, et al., 2020). Quantitative methods include symptoms scales, surveys, or other questionnaires used to describe the target population. Qualitative methods include in-depth interviews with key informants, focus groups, free-list interviews, pile sorting, among others (Cork et al., 2019; Keys et al., 2012). A multistakeholder-perspective is recommended, including clinicians, community

members, potential users of the intervention, and other relevant groups.

We recommend using the consolidated criteria for reporting qualitative research (COREQ, Tong et al., 2007), a 32-items checklist for explicit and comprehensive reporting of qualitative studies. It includes participant selection (i.e., selection, method of approach, sample size, reasons for refusing); the setting for data collection (e.g., home, clinic); the method of data collection (i.e., interview guide, recording, duration), and analysis methods (i.e., how themes were derived from the data).

Once data on the target population is gathered and compiled, interventions are adapted in a bottom-up or top-down approach. And iterative process of adaptation, validation and piloting is recommended (e.g., Shala, Morina, Burchert, et al., 2020). Studies on cultural adaptation of psychological interventions have used focus groups and key informant interviews (Abi Ramia et al., 2018; Garabiles et al., 2019). In addition, "cognitive interviewing" (Willis, 2004) has been used frequently in literature, in which intervention materials are read through and participants "think aloud". Wingood and DiClemente (2008) suggest identifying "topical experts" who can assist in adaptation. And Salamanca-Sanabria et al. (2019) developed an instrument, the Cultural Relevance Questionnaire (CRQ) to support and document the cultural adaptation of psychological interventions.

Do you have any comments on the criterion and the text?

25 Criterion 4. Methods

Criterion 4. Methods

Please rate the relevance of this criterion

- Totally relevant
- Somewhat relevant
- Hardly relevant
- Not at all relevant

26 Criterion 4. Methods

Criterion 4. Methods

Please rate the applicability of this criterion

- Totally applicable

- Somewhat applicable
- Hardly applicable
- Not at all applicable

27 Criterion 5. Definition of the target population

Criterion 5. Definition of the target population

Culture is a complex construct that cannot be reduced to ethnic groups or race. Many different socio-demographic factors may contribute to one's "culture", such as language, religion, age, migration background, refugee status, gender identity, sexual orientation, and socio-economic status, among others (Castro et al., 2010; Sue & Sue, 2015). There is large variety with regard to values and norms within geographically or demographically defined groups (e.g., Fischer & Schwartz, 2011; Resnicow et al., 1999), and people may adopt different "cultural identities" in different contexts (Lehman et al., 2004). Such cultural identities may be influenced.

Therefore, the first step in cultural adaptation is to clearly define the "unit of analysis", i.e., the target population in the psychological trial (Castro et al., 2010). The definition and operationalisation of this unit of analysis should be done along the most important criteria that may have an impact on participants' cultural identity and their psychopathology (Betancourt & López, 1993). The unit of analysis may not always be limited to one particular ethnic, language or even cultural group, i.e. psychological interventions can be culture-sensitive rather than culture-specific. Culture-sensitive interventions may target diverse groups, e.g. migrant populations in high-income countries, and be sensitive to cultural aspects in general rather than adapted to specific features of one particular group (citation: paper in special issue).

Do you agree with this criterion?

- Strongly agree
- Somewhat agree
- Somewhat disagree
- Strongly disagree

28 Criterion 5. Definition of the target population

Criterion 5. Definition of the target population

Culture is a complex construct that cannot be reduced to ethnic groups or race. Many different socio-demographic factors may contribute to one's "culture", such as language, religion, age, migration background, refugee status, gender identity, sexual orientation, and socio-economic status, among others (Castro et al., 2010; Sue & Sue, 2015). There is large variety with regard to values and norms within geographically or demographically defined groups (e.g., Fischer & Schwartz, 2011; Resnicow et al., 1999), and people may adopt different "cultural identities" in different contexts (Lehman et al., 2004). Such cultural identities may be influenced.

Therefore, the first step in cultural adaptation is to clearly define the "unit of analysis", i.e., the target population in

the psychological trial (Castro et al., 2010). The definition and operationalisation of this unit of analysis should be done along the most important criteria that may have an impact on participants' cultural identity and their psychopathology (Betancourt & López, 1993). The unit of analysis may not always be limited to one particular ethnic, language or even cultural group, i.e. psychological interventions can be culture-sensitive rather than culture-specific. Culture-sensitive interventions may target diverse groups, e.g. migrant populations in high-income countries, and be sensitive to cultural aspects in general rather than adapted to specific features of one particular group (citation: paper in special issue).

Do you agree with the description?

- Strongly agree
- Somewhat agree
- Somewhat disagree
- Strongly disagree

29 Criterion 5. Definition of the target population

Criterion 5. Definition of the target population

Culture is a complex construct that cannot be reduced to ethnic groups or race. Many different socio-demographic factors may contribute to one's "culture", such as language, religion, age, migration background, refugee status, gender identity, sexual orientation, and socio-economic status, among others (Castro et al., 2010; Sue & Sue, 2015). There is large variety with regard to values and norms within geographically or demographically defined groups (e.g., Fischer & Schwartz, 2011; Resnicow et al., 1999), and people may adopt different "cultural identities" in different contexts (Lehman et al., 2004). Such cultural identities may be influenced.

Therefore, the first step in cultural adaptation is to clearly define the "unit of analysis", i.e., the target population in the psychological trial (Castro et al., 2010). The definition and operationalisation of this unit of analysis should be done along the most important criteria that may have an impact on participants' cultural identity and their psychopathology (Betancourt & López, 1993). The unit of analysis may not always be limited to one particular ethnic, language or even cultural group, i.e. psychological interventions can be culture-sensitive rather than culture-specific. Culture-sensitive interventions may target diverse groups, e.g. migrant populations in high-income countries, and be sensitive to cultural aspects in general rather than adapted to specific features of one particular group (citation: paper in special issue).

Do you have any comments on the criterion and the text?

30 Criterion 5. Definition of the target population

Criterion 5. Definition of the target population

Please rate the relevance of this criterion

- Totally relevant
- Somewhat relevant
- Hardly relevant
- Not at all relevant

31 Criterion 5. Definition of the target population

Criterion 5. Definition of the target population

Please rate the applicability of this criterion

- Totally applicable
- Somewhat applicable
- Hardly applicable
- Not at all applicable

32 Criterion 6. Target symptoms, syndromes, and needs

Criterion 6. Target symptoms, syndromes, and needs

In a framework for cultural adaptation of psychological interventions that was developed mainly for research settings, Heim and Kohrt (2019) suggest using *cultural concepts of distress* (CCD) as the pivotal point for cultural adaptation. The term CCD has been introduced into the Diagnostic and Statistical Manual of Mental Disorders, fifth edition (DSM-5, American Psychiatric Association, 2013) to describe culturally shaped mental health-related phenomena. CCD encompass *idioms of distress* (Nichter, 1981, 2010), *cultural explanations* (Bhui & Bhugra, 2002), and *cultural syndromes* (Kaiser & Jo Weaver, 2019). Empirical evidence on CCD is emerging in some parts of the world (e.g., Africa, Latin America, East Asia; Kohrt et al., 2014), but there is still a considerable lack of evidence from other regions, e.g. South-Eastern Europe (Shala, Morina, Salis Gross, et al., 2020). In addition to CCD, it is relevant to gather information on particular needs in the target population.

Relevant information for cultural adaptation includes idioms of distress, culture-specific syndromes, specific target symptoms (e.g., anger, pain, or dizziness), core beliefs about human suffering and healing (e.g., fatalism), mental health related stigma, help-seeking behaviours. This information is either gathered through a review of literature, or, if no or insufficient evidence is available, through qualitative research or mixed methods. In their systematic review, Cork et al. (2019) found that most studies used free-list interviews, focus groups, and in-depth interviews with key informants to assess CCD, but quantitative surveys can also be used. In accordance with Criterion 5, the sampling method is important to be considered. Cork et al. (2019) found that in most studies, interviews were done with community samples and not with patients. To assess CCD, it would be relevant to conduct interviews with a clinical sample, to gather their own symptom expressions and cultural explanations (i.e., etiological assumptions).

CCD are distinct from diagnostic categories such as depression, or post-traumatic stress, but in many cases share symptoms with these disorders (e.g., Haroz et al., 2017; Rasmussen et al., 2014). Evidence shows CCD such as spirit possession (Ertl et al., 2011; Patel et al., 1995) in Uganda and Zimbabwe, *dhat* (i.e., semen loss in urine; Gautham et al., 2008) in India, or *hwa-byung*(i.e., fire/projection of [accumulated] anger into the body; Min & Suh, 2010) in Korea were associated with symptoms of psychological distress and mental disorders in general. However, it would be erroneous to conclude that CCD are just variations of the same (universal) underlying constructs across cultural groups. In their systematic review on CCD, Kohrt et al. (2014) argue that higher methodological rigour is needed in order to better understand potential associations and distinctions between CCD and diagnostic categories developed in Western countries.

There are several instruments to examine CCD with patients, e.g., the *Cultural Formulation Interview* in DSM-5 (American Psychiatric Association, 2013), the *Short Explanatory Model Interview* (SEMI, Lloyd et al., 1998)*, the Barts Explanatory Model Inventory* (BEMI, Rüdell et al., 2009) or the *McGill Illness Narrative Interview* (MINI, Groleau et al., 2006). These interviews cover both idioms of distress as well as cultural explanations, along with other relevant aspects such as beliefs about the course of the disorder and help-seeking behaviour. Depending on the available time, the use of such standardised interviews is recommended.

Do you agree with this criterion?

- Strongly agree
- Somewhat agree
- Somewhat disagree
- Strongly disagree

33 Criterion 6. Target symptoms, syndromes, and needs

Criterion 6. Target symptoms, syndromes, and needs

In a framework for cultural adaptation of psychological interventions that was developed mainly for research settings, Heim and Kohrt (2019) suggest using *cultural concepts of distress* (CCD) as the pivotal point for cultural adaptation. The term CCD has been introduced into the Diagnostic and Statistical Manual of Mental Disorders, fifth edition (DSM-5, American Psychiatric Association, 2013) to describe culturally shaped mental health-related phenomena. CCD encompass *idioms of distress* (Nichter, 1981, 2010), *cultural explanations* (Bhui & Bhugra, 2002), and *cultural syndromes* (Kaiser & Jo Weaver, 2019). Empirical evidence on CCD is emerging in some parts of the world (e.g., Africa, Latin America, East Asia; Kohrt et al., 2014), but there is still a considerable lack of evidence from other regions, e.g. South-Eastern Europe (Shala, Morina, Salis Gross, et al., 2020). In addition to CCD, it is relevant to gather information on particular needs in the target population.

Relevant information for cultural adaptation includes idioms of distress, culture-specific syndromes, specific target symptoms (e.g., anger, pain, or dizziness), core beliefs about human suffering and healing (e.g., fatalism), mental health related stigma, help-seeking behaviours. This information is either gathered through a review of literature, or, if no or insufficient evidence is available, through qualitative research or mixed methods. In their systematic review, Cork et al. (2019) found that most studies used free-list interviews, focus groups, and in-depth interviews with key informants to assess CCD, but quantitative surveys can also be used. In accordance with Criterion 5, the sampling method is important to be considered. Cork et al. (2019) found that in most studies, interviews were

done with community samples and not with patients. To assess CCD, it would be relevant to conduct interviews with a clinical sample, to gather their own symptom expressions and cultural explanations (i.e., etiological assumptions).

CCD are distinct from diagnostic categories such as depression, or post-traumatic stress, but in many cases share symptoms with these disorders (e.g., Haroz et al., 2017; Rasmussen et al., 2014). Evidence shows CCD such as spirit possession (Ertl et al., 2011; Patel et al., 1995) in Uganda and Zimbabwe, *dhat* (i.e., semen loss in urine; Gautham et al., 2008) in India, or *hwa-byung*(i.e., fire/projection of [accumulated] anger into the body; Min & Suh, 2010) in Korea were associated with symptoms of psychological distress and mental disorders in general. However, it would be erroneous to conclude that CCD are just variations of the same (universal) underlying constructs across cultural groups. In their systematic review on CCD, Kohrt et al. (2014) argue that higher methodological rigour is needed in order to better understand potential associations and distinctions between CCD and diagnostic categories developed in Western countries.

There are several instruments to examine CCD with patients, e.g., the *Cultural Formulation Interview* in DSM-5 (American Psychiatric Association, 2013), the *Short Explanatory Model Interview* (SEMI, Lloyd et al., 1998)*, the Barts Explanatory Model Inventory* (BEMI, Rüdell et al., 2009) or the *McGill Illness Narrative Interview* (MINI, Groleau et al., 2006). These interviews cover both idioms of distress as well as cultural explanations, along with other relevant aspects such as beliefs about the course of the disorder and help-seeking behaviour. Depending on the available time, the use of such standardised interviews is recommended.

Do you agree with the description?

- ○ Strongly agree
- ○ Somewhat agree
- ○ Somewhat disagree
- ○ Strongly disagree

34 Criterion 6. Target symptoms, syndromes, and needs

Criterion 6. Target symptoms, syndromes, and needs

In a framework for cultural adaptation of psychological interventions that was developed mainly for research settings, Heim and Kohrt (2019) suggest using *cultural concepts of distress* (CCD) as the pivotal point for cultural adaptation. The term CCD has been introduced into the Diagnostic and Statistical Manual of Mental Disorders, fifth edition (DSM-5, American Psychiatric Association, 2013) to describe culturally shaped mental health-related phenomena. CCD encompass *idioms of distress* (Nichter, 1981, 2010), *cultural explanations* (Bhui & Bhugra, 2002), and *cultural syndromes* (Kaiser & Jo Weaver, 2019). Empirical evidence on CCD is emerging in some parts of the world (e.g., Africa, Latin America, East Asia; Kohrt et al., 2014), but there is still a considerable lack of evidence from other regions, e.g. South-Eastern Europe (Shala, Morina, Salis Gross, et al., 2020). In addition to CCD, it is relevant to gather information on particular needs in the target population.

Relevant information for cultural adaptation includes idioms of distress, culture-specific syndromes, specific target symptoms (e.g., anger, pain, or dizziness), core beliefs about human suffering and healing (e.g., fatalism), mental health related stigma, help-seeking behaviours. This information is either gathered through a review of literature, or, if no or insufficient evidence is available, through qualitative research or mixed methods. In their systematic

review, Cork et al. (2019) found that most studies used free-list interviews, focus groups, and in-depth interviews with key informants to assess CCD, but quantitative surveys can also be used. In accordance with Criterion 5, the sampling method is important to be considered. Cork et al. (2019) found that in most studies, interviews were done with community samples and not with patients. To assess CCD, it would be relevant to conduct interviews with a clinical sample, to gather their own symptom expressions and cultural explanations (i.e., etiological assumptions).

CCD are distinct from diagnostic categories such as depression, or post-traumatic stress, but in many cases share symptoms with these disorders (e.g., Haroz et al., 2017; Rasmussen et al., 2014). Evidence shows CCD such as spirit possession (Ertl et al., 2011; Patel et al., 1995) in Uganda and Zimbabwe, *dhat* (i.e., semen loss in urine; Gautham et al., 2008) in India, or *hwa-byung*(i.e., fire/projection of [accumulated] anger into the body; Min & Suh, 2010) in Korea were associated with symptoms of psychological distress and mental disorders in general. However, it would be erroneous to conclude that CCD are just variations of the same (universal) underlying constructs across cultural groups. In their systematic review on CCD, Kohrt et al. (2014) argue that higher methodological rigour is needed in order to better understand potential associations and distinctions between CCD and diagnostic categories developed in Western countries.

There are several instruments to examine CCD with patients, e.g., the *Cultural Formulation Interview* in DSM-5 (American Psychiatric Association, 2013), the *Short Explanatory Model Interview* (SEMI, Lloyd et al., 1998)*, the Barts Explanatory Model Inventory* (BEMI, Rüdell et al., 2009) or the *McGill Illness Narrative Interview* (MINI, Groleau et al., 2006). These interviews cover both idioms of distress as well as cultural explanations, along with other relevant aspects such as beliefs about the course of the disorder and help-seeking behaviour. Depending on the available time, the use of such standardised interviews is recommended.

Do you have any comments on the criterion and the text?

35 Criterion 6. Target symptoms, syndromes, and needs

Criterion 6. Target symptoms, syndromes, and needs

Please rate the relevance of this criterion

- ◯ Totally relevant
- ◯ Somewhat relevant
- ◯ Hardly relevant
- ◯ Not at all relevant

36 Criterion 6. Target symptoms, syndromes, and needs

Criterion 6. Target symptoms, syndromes, and needs

Please rate the applicability of this criterion

- Totally applicable
- Somewhat applicable
- Hardly applicable
- Not at all applicable

37 C) Intervention

C) Intervention

In literature, cultural adaptation of psychological interventions most often implicitly refers to the top-down approach, in which existing psychological interventions developed for one cultural group are adapted for another one (Hall et al., 2016; Hwang, 2006). There is little evidence on psychological interventions adapted in a bottom-up approach to address culture-specific symptoms and syndromes. Our suggested reporting criteria are applicable to both approaches and may thereby contribute to an increase in studies doing bottom-up cultural adaptation.

One might argue that the development of new interventions to target specific CCD does not fall under "adaptation". We counter this argument by stating that psychological interventions for the treatment of distress and mental disorders are a Western concept by themselves, as is the empirical evaluation of such interventions through randomised controlled trials. Therefore, the present reporting criteria are applicable not only for trials testing culturally adapted versions of existing interventions, but also newly developed interventions and intervention components that aim to target CCD among culturally diverse groups.

Psychological interventions and trials to evaluate them share a common set of features, which have been classified by Singla et al. (2017) into four categories: Who (i.e., provider); What (i.e., treatment components); Where (i.e., treatment setting); and How (i.e., training, supervision, treatment delivery). Treatment components are further divided into: i) specific elements that are based on theoretical psychological models; ii) unspecific elements that are commonly shared by interventions of different theoretical backgrounds; and iii) therapeutic techniques that aim to transmit specific and unspecific elements.

This taxonomy provides a helpful grid to support the cultural adaptation of intervention, as it specifies the different levels of an intervention. Other frameworks (e.g., Bernal et al., 1995; Bernal & Sáez-Santiago, 2006) have listed elements for cultural adaptation without putting them into a functional relationship. Accordingly, we structured our reporting criteria along this taxonomy.

Do you have any comments on the text?

38 Criterion 7. Specific elements

Criterion 7. Specific elements

Most psychological trials have used manuals or protocols as unit of analysis (Chorpita & Daleiden, 2009). Manuals most often focus on one particular diagnosis and use a series of elements for the treatment of this disorder (e.g., psychoeducation, exposition, cognitive restructuring, relapse prevention) for their treatment. By contrast, transdiagnostic interventions combine treatment elements to address a broader symptom spectrum instead of one particular diagnosis, with promising effect sizes (Newby et al., 2015). As an example, the Common Elements Treatment Approach (CETA, Murray et al., 2014), applies evidence-based treatment elements depending on the specific symptomatology of the patient. Another example is Problem Management Plus (PM+) developed by World Health Organisation (WHO), which combines evidence-based treatment elements in a transdiagnostic, low-intensity intervention (Dawson et al., 2015).

Considering the fact that CCD do not necessarily correspond to Western diagnostic categories (see above), the selection of a manual or specific treatment elements is ideally dovetailed with evidence on CCD in the target group. Such specific elements, even if evidence-based, might sometimes not or only partly correspond to the most salient symptoms in the target group. As an example, behavioural activation is based on the theoretical assumption that inertia and avoidance are important maintaining factors in depression (Ferster, 1973; Lewinsohn, 1974; Veale, 2008), and behavioural activation a key mechanisms of action in depression treatment. However, a systematic review of qualitative studies about depression around the world demonstrated that “the majority of study populations did not raise problems with daily functioning as part of their subjective experiences of depression” (Haroz et al., 2017, p. 160). Instead, irritability, anger, and pain were prominent symptoms often related to depression. This raises the question whether behavioural activation is the first choice as a specific element to treat depression among diverse cultural and ethnic groups, or whether other interventions, e.g. stress management and emotion regulation skills, would be more effective.

Studies with diverse ethnic and cultural groups sometimes provide reasons for the choice of specific components, e.g. arguing that behavioural activation is easier to explain than cognitive techniques , especially if the intervention is provided by lay helpers (Dawson et al., 2015). Two studies (citation: paper in special issue) showed their reasons for choosing/omitting/adapting problem management as specific treatment elements in their interventions with refugees. Similarly, Carswell et al. (2018) developed a mobile- and Internet-based intervention (called Step-by-Step) for the treatment of depression among populations affected by adversities in low- and middle-income countries. Although Step-by-Step was based on the manual called Problem Management Plus (Dawson et al., 2015), problem management was omitted from the Step-by-Step intervention because it was assumed to be difficult to explain through a self-help intervention provided through technology.

For psychological trials with diverse ethnic and cultural groups, it may be relevant to provide some empirically or theoretically based rationale for the selection, omission or adaptation of specific elements. This rationale may either be related to existing evidence on CCD in the target population, or to other aspects such as literacy levels, mode of delivery (see below), or cultural values (citation: paper in special issue). In addition, it is relevant to report on specific elements that are left unchanged after a process of formative research and reflection (citation: paper in

special issue). In other words, explicit decisions to leave specific elements unchanged should be reported, as well.

Do you agree with this criterion?

- Strongly agree
- Somewhat agree
- Somewhat disagree
- Strongly disagree

39 Criterion 7. Specific elements

Criterion 7. Specific elements

Most psychological trials have used manuals or protocols as unit of analysis (Chorpita & Daleiden, 2009). Manuals most often focus on one particular diagnosis and use a series of elements for the treatment of this disorder (e.g., psychoeducation, exposition, cognitive restructuring, relapse prevention) for their treatment. By contrast, transdiagnostic interventions combine treatment elements to address a broader symptom spectrum instead of one particular diagnosis, with promising effect sizes (Newby et al., 2015). As an example, the Common Elements Treatment Approach (CETA, Murray et al., 2014), applies evidence-based treatment elements depending on the specific symptomatology of the patient. Another example is Problem Management Plus (PM+) developed by World Health Organisation (WHO), which combines evidence-based treatment elements in a transdiagnostic, low-intensity intervention (Dawson et al., 2015).

Considering the fact that CCD do not necessarily correspond to Western diagnostic categories (see above), the selection of a manual or specific treatment elements is ideally dovetailed with evidence on CCD in the target group. Such specific elements, even if evidence-based, might sometimes not or only partly correspond to the most salient symptoms in the target group. As an example, behavioural activation is based on the theoretical assumption that inertia and avoidance are important maintaining factors in depression (Ferster, 1973; Lewinsohn, 1974; Veale, 2008), and behavioural activation a key mechanisms of action in depression treatment. However, a systematic review of qualitative studies about depression around the world demonstrated that “the majority of study populations did not raise problems with daily functioning as part of their subjective experiences of depression” (Haroz et al., 2017, p. 160). Instead, irritability, anger, and pain were prominent symptoms often related to depression. This raises the question whether behavioural activation is the first choice as a specific element to treat depression among diverse cultural and ethnic groups, or whether other interventions, e.g. stress management and emotion regulation skills, would be more effective.

Studies with diverse ethnic and cultural groups sometimes provide reasons for the choice of specific components, e.g. arguing that behavioural activation is easier to explain than cognitive techniques , especially if the intervention is provided by lay helpers (Dawson et al., 2015). Two studies (citation: paper in special issue) showed their reasons for choosing/omitting/adapting problem management as specific treatment elements in their interventions with refugees. Similarly, Carswell et al. (2018) developed a mobile- and Internet-based intervention (called Step-by-Step) for the treatment of depression among populations affected by adversities in low- and middle-income countries. Although Step-by-Step was based on the manual called Problem Management Plus (Dawson et al., 2015), problem management was omitted from the Step-by-Step intervention because it was assumed to be difficult to explain through a self-help intervention provided through technology.

For psychological trials with diverse ethnic and cultural groups, it may be relevant to provide some empirically or theoretically based rationale for the selection, omission or adaptation of specific elements. This rationale may either be related to existing evidence on CCD in the target population, or to other aspects such as literacy levels, mode of delivery (see below), or cultural values (citation: paper in special issue). In addition, it is relevant to report on specific elements that are left unchanged after a process of formative research and reflection (citation: paper in special issue). In other words, explicit decisions to leave specific elements unchanged should be reported, as well.

Do you agree with the description?

- Strongly agree
- Somewhat agree
- Somewhat disagree
- Strongly disagree

40 Criterion 7. Specific elements

Criterion 7. Specific elements

Most psychological trials have used manuals or protocols as unit of analysis (Chorpita & Daleiden, 2009). Manuals most often focus on one particular diagnosis and use a series of elements for the treatment of this disorder (e.g., psychoeducation, exposition, cognitive restructuring, relapse prevention) for their treatment. By contrast, transdiagnostic interventions combine treatment elements to address a broader symptom spectrum instead of one particular diagnosis, with promising effect sizes (Newby et al., 2015). As an example, the Common Elements Treatment Approach (CETA, Murray et al., 2014), applies evidence-based treatment elements depending on the specific symptomatology of the patient. Another example is Problem Management Plus (PM+) developed by World Health Organisation (WHO), which combines evidence-based treatment elements in a transdiagnostic, low-intensity intervention (Dawson et al., 2015).

Considering the fact that CCD do not necessarily correspond to Western diagnostic categories (see above), the selection of a manual or specific treatment elements is ideally dovetailed with evidence on CCD in the target group. Such specific elements, even if evidence-based, might sometimes not or only partly correspond to the most salient symptoms in the target group. As an example, behavioural activation is based on the theoretical assumption that inertia and avoidance are important maintaining factors in depression (Ferster, 1973; Lewinsohn, 1974; Veale, 2008), and behavioural activation a key mechanisms of action in depression treatment. However, a systematic review of qualitative studies about depression around the world demonstrated that “the majority of study populations did not raise problems with daily functioning as part of their subjective experiences of depression” (Haroz et al., 2017, p. 160). Instead, irritability, anger, and pain were prominent symptoms often related to depression. This raises the question whether behavioural activation is the first choice as a specific element to treat depression among diverse cultural and ethnic groups, or whether other interventions, e.g. stress management and emotion regulation skills, would be more effective.

Studies with diverse ethnic and cultural groups sometimes provide reasons for the choice of specific components, e.g. arguing that behavioural activation is easier to explain than cognitive techniques , especially if the intervention is provided by lay helpers (Dawson et al., 2015). Two studies (citation: paper in special issue) showed their reasons for choosing/omitting/adapting problem management as specific treatment elements in their interventions with refugees. Similarly, Carswell et al. (2018) developed a mobile- and Internet-based intervention (called Step-by-Step) for the treatment of depression among populations affected by adversities in low- and middle-income

countries. Although Step-by-Step was based on the manual called Problem Management Plus (Dawson et al., 2015), problem management was omitted from the Step-by-Step intervention because it was assumed to be difficult to explain through a self-help intervention provided through technology.

For psychological trials with diverse ethnic and cultural groups, it may be relevant to provide some empirically or theoretically based rationale for the selection, omission or adaptation of specific elements. This rationale may either be related to existing evidence on CCD in the target population, or to other aspects such as literacy levels, mode of delivery (see below), or cultural values (citation: paper in special issue). In addition, it is relevant to report on specific elements that are left unchanged after a process of formative research and reflection (citation: paper in special issue). In other words, explicit decisions to leave specific elements unchanged should be reported, as well.

Do you have any comments on the criterion and the text?

41 Criterion 7. Specific elements

Criterion 7. Specific elements

Please rate the relevance of this criterion

- ○ Totally relevant
- ○ Somewhat relevant
- ○ Hardly relevant
- ○ Not at all relevant

42 Criterion 7. Specific elements

Criterion 7. Specific elements

Please rate the applicability of this criterion

- ○ Totally applicable
- ○ Somewhat applicable
- ○ Hardly applicable
- ○ Not at all applicable

43 Criterion 8. Unspecific elements and therapeutic techniques

Criterion 8. Unspecific elements and therapeutic techniques

Unspecific elements refer to components that are universal to all treatments, also known as “common factors” (Cuijpers et al., 2019; Wampold, 2007). One important common factor is the provision of a convincing treatment rationale. Psychological interventions ideally provide explanations that differ from the patient’s views, but that are not too discrepant from the patient’s intuitive assumptions as to be rejected (Wampold, 2007). For treatment adherence and compliance, it is vital that patients understand and to some point share the rationale behind the treatment.

The treatment rationale is ideally dovetailed with cultural explanations and idioms of distress that are part of CCD (Hwang, 2006; Rathod et al., 2019). Cork et al. (2019) conducted a systematic review on how to integrate CCD, and particularly idioms of distress, into existing mental health assessments and interventions. CCD may include beliefs and assumptions that require to be challenged when providing the treatment rationale. As an example, Reich, Zürn, and Mewes (2019) developed a web-based intervention to enhance motivation for psychotherapy among Turkish immigrants in Germany who held fatalistic beliefs. In addition, it may be relevant to consider culturally-specific notions of stigma, and the way how mental health-related stigma threatens the life domains that “matter most” (Yang et al., 2014) to members of a specific cultural group (e.g., marriage, employment, social networks).

Reporting on cultural adaptation of unspecific components is recommended, especially to enhance empirical evidence on the effect of this adaptation. As an example, if the treatment rationale is culturally adapted, while leaving all other components of the intervention unchanged, this may indicate that the rationale itself is a mechanism of action in the corresponding treatment (Heim et al., 2020). In addition, we recommend to report on the reflections that have guided the choice, omission, or adaptation of therapeutic techniques, such as role-playing, goal setting, homework, or behavioural experiments (Singla et al., 2017).

Do you agree with this criterion?

- ○ Strongly agree
- ○ Somewhat agree
- ○ Somewhat disagree
- ○ Strongly disagree

44 Criterion 8. Unspecific elements and therapeutic techniques

Criterion 8. Unspecific elements and therapeutic techniques

Unspecific elements refer to components that are universal to all treatments, also known as “common factors” (Cuijpers et al., 2019; Wampold, 2007). One important common factor is the provision of a convincing treatment rationale. Psychological interventions ideally provide explanations that differ from the patient’s views, but that are not too discrepant from the patient’s intuitive assumptions as to be rejected (Wampold, 2007). For treatment adherence and compliance, it is vital that patients understand and to some point share the rationale behind the treatment.

The treatment rationale is ideally dovetailed with cultural explanations and idioms of distress that are part of CCD (Hwang, 2006; Rathod et al., 2019). Cork et al. (2019) conducted a systematic review on how to integrate CCD, and particularly idioms of distress, into existing mental health assessments and interventions. CCD may include beliefs and assumptions that require to be challenged when providing the treatment rationale. As an example, Reich, Zürn, and Mewes (2019) developed a web-based intervention to enhance motivation for psychotherapy among Turkish immigrants in Germany who held fatalistic beliefs. In addition, it may be relevant to consider culturally-specific notions of stigma, and the way how mental health-related stigma threatens the life domains that "matter most" (Yang et al., 2014) to members of a specific cultural group (e.g., marriage, employment, social networks).

Reporting on cultural adaptation of unspecific components is recommended, especially to enhance empirical evidence on the effect of this adaptation. As an example, if the treatment rationale is culturally adapted, while leaving all other components of the intervention unchanged, this may indicate that the rationale itself is a mechanism of action in the corresponding treatment (Heim et al., 2020). In addition, we recommend to report on the reflections that have guided the choice, omission, or adaptation of therapeutic techniques, such as role-playing, goal setting, homework, or behavioural experiments (Singla et al., 2017).

Do you agree with the description?

- ○ Strongly agree
- ○ Somewhat agree
- ○ Somewhat disagree
- ○ Strongly disagree

45 Criterion 8. Unspecific elements and therapeutic techniques

Criterion 8. Unspecific elements and therapeutic techniques

Unspecific elements refer to components that are universal to all treatments, also known as "common factors" (Cuijpers et al., 2019; Wampold, 2007). One important common factor is the provision of a convincing treatment rationale. Psychological interventions ideally provide explanations that differ from the patient's views, but that are not too discrepant from the patient's intuitive assumptions as to be rejected (Wampold, 2007). For treatment adherence and compliance, it is vital that patients understand and to some point share the rationale behind the treatment.

The treatment rationale is ideally dovetailed with cultural explanations and idioms of distress that are part of CCD (Hwang, 2006; Rathod et al., 2019). Cork et al. (2019) conducted a systematic review on how to integrate CCD, and particularly idioms of distress, into existing mental health assessments and interventions. CCD may include beliefs and assumptions that require to be challenged when providing the treatment rationale. As an example, Reich, Zürn, and Mewes (2019) developed a web-based intervention to enhance motivation for psychotherapy among Turkish immigrants in Germany who held fatalistic beliefs. In addition, it may be relevant to consider culturally-specific notions of stigma, and the way how mental health-related stigma threatens the life domains that "matter most" (Yang et al., 2014) to members of a specific cultural group (e.g., marriage, employment, social networks).

Reporting on cultural adaptation of unspecific components is recommended, especially to enhance empirical

evidence on the effect of this adaptation. As an example, if the treatment rationale is culturally adapted, while leaving all other components of the intervention unchanged, this may indicate that the rationale itself is a mechanism of action in the corresponding treatment (Heim et al., 2020). In addition, we recommend to report on the reflections that have guided the choice, omission, or adaptation of therapeutic techniques, such as role-playing, goal setting, homework, or behavioural experiments (Singla et al., 2017).

Do you have any comments on the criterion and the text?

46 Criterion 8. Unspecific elements and therapeutic techniques

Criterion 8. Unspecific elements and therapeutic techniques

Please rate the relevance of this criterion

- ○ Totally relevant
- ○ Somewhat relevant
- ○ Hardly relevant
- ○ Not at all relevant

47 Criterion 8. Unspecific elements and therapeutic techniques

Criterion 8. Unspecific elements and therapeutic techniques

Please rate the applicability of this criterion

- ○ Totally applicable
- ○ Somewhat applicable
- ○ Hardly applicable
- ○ Not at all applicable

48 Criterion 9. Surface adaptations

Criterion 9. Surface adaptations

Surface structure adaptations aim to enhance acceptance of an intervention through matching materials, channels

and settings to the target population (Resnicow et al., 1999). Such surface adaptations correspond to the How and Where in the taxonomy suggested by Singla et al. (2017). There is much evidence on such surface adaptations of psychological interventions (Chowdhary et al., 2014; Chu & Leino, 2017; Harper Shehadeh et al., 2016).

Cultural and contextual factors may determine the channels through which the treatment components are provided, e.g. group-based as opposed to individual treatment (Epping-Jordan et al., 2016; Sangraula et al., 2018; Verdeli et al., 2003), or internet-based interventions (Naslund et al., 2017) that are increasingly tested and applied among diverse ethnic and cultural groups. Reporting should include considerations that have been made with regard to such different modes of delivery.

For self-help interventions such as internet- and smartphone-based programmes, an additional process is required of creating software functions and user-interfaces that match the chosen content and therapeutic techniques as well as characteristics of the intended users. Ideally, this process is conducted in a systematic manner using design thinking and user-centred design approaches (Mummah et al., 2016). These approaches utilize interviews, focus groups, mock-ups, and interactive prototypes in an iterative manner to optimize usability and the fit with the needs, expectations, and technical proficiency of the target group (e.g., Burchert et al., 2019).

Most interventions (both self-help and face-to-face) include materials such as texts, illustrations, case examples, flyers, audio files, videos, etc. Several standards exist for the translation of assessments and materials. van Ommeren et al. (1999) suggest to use a translation monitoring form. Several studies report that it is often difficult to draw the line between translation and adaptation, as these two are closely intertwined (Ramaiya et al., 2017; Shala et al., accepted). For pragmatic reasons, it is often not possible to document all the decisions that were made during the process of translation and language editing, especially if the decisions are merely questions of style or grammar. However, some decisions might be relevant to be documented in the cultural adaptation monitoring sheet. As an example, metaphors are often culture-specific and cannot be translated literally (Rechsteiner et al., 2020). It might therefore make sense to report on how specific metaphors in the intervention were translated or adapted.

Do you agree with this criterion?

- Strongly agree
- Somewhat agree
- Somewhat disagree
- Strongly disagree

49 Criterion 9. Surface adaptations

Criterion 9. Surface adaptations

Surface structure adaptations aim to enhance acceptance of an intervention through matching materials, channels and settings to the target population (Resnicow et al., 1999). Such surface adaptations correspond to the How and Where in the taxonomy suggested by Singla et al. (2017). There is much evidence on such surface adaptations of psychological interventions (Chowdhary et al., 2014; Chu & Leino, 2017; Harper Shehadeh et al., 2016).

Cultural and contextual factors may determine the channels through which the treatment components are

provided, e.g. group-based as opposed to individual treatment (Epping-Jordan et al., 2016; Sangraula et al., 2018; Verdeli et al., 2003), or internet-based interventions (Naslund et al., 2017) that are increasingly tested and applied among diverse ethnic and cultural groups. Reporting should include considerations that have been made with regard to such different modes of delivery.

For self-help interventions such as internet- and smartphone-based programmes, an additional process is required of creating software functions and user-interfaces that match the chosen content and therapeutic techniques as well as characteristics of the intended users. Ideally, this process is conducted in a systematic manner using design thinking and user-centred design approaches (Mummah et al., 2016). These approaches utilize interviews, focus groups, mock-ups, and interactive prototypes in an iterative manner to optimize usability and the fit with the needs, expectations, and technical proficiency of the target group (e.g., Burchert et al., 2019).

Most interventions (both self-help and face-to-face) include materials such as texts, illustrations, case examples, flyers, audio files, videos, etc. Several standards exist for the translation of assessments and materials. van Ommeren et al. (1999) suggest to use a translation monitoring form. Several studies report that it is often difficult to draw the line between translation and adaptation, as these two are closely intertwined (Ramaiya et al., 2017; Shala et al., accepted). For pragmatic reasons, it is often not possible to document all the decisions that were made during the process of translation and language editing, especially if the decisions are merely questions of style or grammar. However, some decisions might be relevant to be documented in the cultural adaptation monitoring sheet. As an example, metaphors are often culture-specific and cannot be translated literally (Rechsteiner et al., 2020). It might therefore make sense to report on how specific metaphors in the intervention were translated or adapted.

Do you agree with the description?

- Strongly agree
- Somewhat agree
- Somewhat disagree
- Strongly disagree

50 Criterion 9. Surface adaptations

Criterion 9. Surface adaptations

Surface structure adaptations aim to enhance acceptance of an intervention through matching materials, channels and settings to the target population (Resnicow et al., 1999). Such surface adaptations correspond to the How and Where in the taxonomy suggested by Singla et al. (2017). There is much evidence on such surface adaptations of psychological interventions (Chowdhary et al., 2014; Chu & Leino, 2017; Harper Shehadeh et al., 2016).

Cultural and contextual factors may determine the channels through which the treatment components are provided, e.g. group-based as opposed to individual treatment (Epping-Jordan et al., 2016; Sangraula et al., 2018; Verdeli et al., 2003), or internet-based interventions (Naslund et al., 2017) that are increasingly tested and applied among diverse ethnic and cultural groups. Reporting should include considerations that have been made with regard to such different modes of delivery.

For self-help interventions such as internet- and smartphone-based programmes, an additional process is required of creating software functions and user-interfaces that match the chosen content and therapeutic techniques as well as characteristics of the intended users. Ideally, this process is conducted in a systematic manner using design thinking and user-centred design approaches (Mummah et al., 2016). These approaches utilize interviews, focus groups, mock-ups, and interactive prototypes in an iterative manner to optimize usability and the fit with the needs, expectations, and technical proficiency of the target group (e.g., Burchert et al., 2019).

Most interventions (both self-help and face-to-face) include materials such as texts, illustrations, case examples, flyers, audio files, videos, etc. Several standards exist for the translation of assessments and materials. van Ommeren et al. (1999) suggest to use a translation monitoring form. Several studies report that it is often difficult to draw the line between translation and adaptation, as these two are closely intertwined (Ramaiya et al., 2017; Shala et al., accepted). For pragmatic reasons, it is often not possible to document all the decisions that were made during the process of translation and language editing, especially if the decisions are merely questions of style or grammar. However, some decisions might be relevant to be documented in the cultural adaptation monitoring sheet. As an example, metaphors are often culture-specific and cannot be translated literally (Rechsteiner et al., 2020). It might therefore make sense to report on how specific metaphors in the intervention were translated or adapted.

Do you have any comments on the criterion and the text?

51 Criterion 9. Surface adaptations

Criterion 9. Surface adaptations

Please rate the relevance of this criterion

- Totally relevant
- Somewhat relevant
- Hardly relevant
- Not at all relevant

52 Criterion 9. Surface adaptations

Criterion 9. Surface adaptations

Please rate the applicability of this criterion

- Totally applicable

○ Somewhat applicable

○ Hardly applicable

○ Not at all applicable

53 D) Measuring outcomes

D) Measuring outcomes

As outlined above, there is considerable cultural variation in symptom expression. In clinical trials testing psychological interventions among diverse ethnic and cultural groups, it is vital to account for this cultural validation by using validated instruments.

Do you have any comments on the text?

54 Criterion 10. Questionnaires

Criterion 10. Questionnaires

There are standard criteria for the translation, adaptation and validation of questionnaires. As an example, Wild et al. (2005) and van Ommeren et al. (1999) provide principles of good practice for the translation and cultural adaptation process for patient-reported outcomes. In addition, standard psychometric methods for the cross-cultural validation of questionnaires have been developed (e.g., Byrne et al., 1989; Milfont & Fischer, 2010; Vandenberg & Lance, 2000).

The validity of questionnaires can be enhanced by incorporating CCD, and particularly idioms of distress. As an example, Hinton et al. (2019) suggest adding items such as ‘‘thinking a lot,’’ ‘‘weak heart,’’ or ‘‘blurry vision’’ to the assessments of depression and anxiety among Cambodian patients, as these symptoms are salient for psychological distress in this particular group. In their systematic review, Cork et al. (2019) found that some studies integrated idioms of distress into existing questionnaires while others developed new assessments for the groups where CCD were gathered.

Another option is the use of person-centred outcome measures such as the Psychological Outcome Profiles instrument (PSYCHLOPS, Ashworth et al., 2004). The PSYCHLOPS consisting of four scored questions and three domains: problems (2 questions), functioning (1 question) and wellbeing (1 question). Participants are asked to give free text responses to the problem and function domains. Thereafter, they are asked to indicate how much they are affected by the problems they mentioned. The pre- and post-therapy versions of PSYCHLOPS consist of the same four questions but the post-therapy version adds an overall evaluation of how they feel compared to the start of the programme (ranging from much better to much worse). The PSYCHLOPS has been validated in several countries (e.g., Czachowski et al., 2011; Héðinsson et al., 2013).

Do you agree with this criterion?

- Strongly agree
- Somewhat agree
- Somewhat disagree
- Strongly disagree

55 Criterion 10. Questionnaires

Criterion 10. Questionnaires

There are standard criteria for the translation, adaptation and validation of questionnaires. As an example, Wild et al. (2005) and van Ommeren et al. (1999) provide principles of good practice for the translation and cultural adaptation process for patient-reported outcomes. In addition, standard psychometric methods for the cross-cultural validation of questionnaires have been developed (e.g., Byrne et al., 1989; Milfont & Fischer, 2010; Vandenberg & Lance, 2000).

The validity of questionnaires can be enhanced by incorporating CCD, and particularly idioms of distress. As an example, Hinton et al. (2019) suggest adding items such as ‘‘thinking a lot,’’ ‘‘weak heart,’’ or ‘‘blurry vision’’ to the assessments of depression and anxiety among Cambodian patients, as these symptoms are salient for psychological distress in this particular group. In their systematic review, Cork et al. (2019) found that some studies integrated idioms of distress into existing questionnaires while others developed new assessments for the groups where CCD were gathered.

Another option is the use of person-centred outcome measures such as the Psychological Outcome Profiles instrument (PSYCHLOPS, Ashworth et al., 2004). The PSYCHLOPS consisting of four scored questions and three domains: problems (2 questions), functioning (1 question) and wellbeing (1 question). Participants are asked to give free text responses to the problem and function domains. Thereafter, they are asked to indicate how much they are affected by the problems they mentioned. The pre- and post-therapy versions of PSYCHLOPS consist of the same four questions but the post-therapy version adds an overall evaluation of how they feel compared to the start of the programme (ranging from much better to much worse). The PSYCHLOPS has been validated in several countries (e.g., Czachowski et al., 2011; Héðinsson et al., 2013).

Do you agree with the description?

- Strongly agree
- Somewhat agree
- Somewhat disagree
- Strongly disagree

56 Criterion 10. Questionnaires

Criterion 10. Questionnaires

There are standard criteria for the translation, adaptation and validation of questionnaires. As an example, Wild et al. (2005) and van Ommeren et al. (1999) provide principles of good practice for the translation and cultural adaptation process for patient-reported outcomes. In addition, standard psychometric methods for the cross-cultural validation of questionnaires have been developed (e.g., Byrne et al., 1989; Milfont & Fischer, 2010; Vandenberg & Lance, 2000).

The validity of questionnaires can be enhanced by incorporating CCD, and particularly idioms of distress. As an example, Hinton et al. (2019) suggest adding items such as ‘‘thinking a lot,’’ ‘‘weak heart,’’ or ‘‘blurry vision’’ to the assessments of depression and anxiety among Cambodian patients, as these symptoms are salient for psychological distress in this particular group. In their systematic review, Cork et al. (2019) found that some studies integrated idioms of distress into existing questionnaires while others developed new assessments for the groups where CCD were gathered.

Another option is the use of person-centred outcome measures such as the Psychological Outcome Profiles instrument (PSYCHLOPS, Ashworth et al., 2004). The PSYCHLOPS consisting of four scored questions and three domains: problems (2 questions), functioning (1 question) and wellbeing (1 question). Participants are asked to give free text responses to the problem and function domains. Thereafter, they are asked to indicate how much they are affected by the problems they mentioned. The pre- and post-therapy versions of PSYCHLOPS consist of the same four questions but the post-therapy version adds an overall evaluation of how they feel compared to the start of the programme (ranging from much better to much worse). The PSYCHLOPS has been validated in several countries (e.g., Czachowski et al., 2011; Héðinsson et al., 2013).

Do you have any comments on the criterion and the text?

57 Criterion 10. Questionnaires

Criterion 10. Questionnaires

Please rate the relevance of this criterion

- ○ Totally relevant
- ○ Somewhat relevant
- ○ Hardly relevant
- ○ Not at all relevant

58 Criterion 10. Questionnaires

Criterion 10. Questionnaires

Please rate the applicability of this criterion

- ○ Totally applicable
- ○ Somewhat applicable
- ○ Hardly applicable
- ○ Not at all applicable

59 Criterion 11. Clinical interviews

Criterion 11. Clinical interviews

Most trials use self-report questionnaires as their primary outcome measure. Clinical interviews are of course more labour-intensive, but the diagnostic accuracy might be higher (Ferrari et al., 2013), especially in non-Western cultural contexts. If the planned outcome measure for the psychological trial is a clinical interview, it is recommended to integrate a culture-sensitive interview, such as the *Cultural Formulation Interview* in DSM-5 (American Psychiatric Association, 2013). Training interviewers in culture-sensitive assessments is important, in order to avoid misdiagnosis. And it is relevant to report on interviewer training and interrater reliability with regard to cultural competence.

Do you agree with this criterion?

- ○ Strongly agree
- ○ Somewhat agree
- ○ Somewhat disagree
- ○ Strongly disagree

60 Criterion 11. Clinical interviews

Criterion 11. Clinical interviews

Most trials use self-report questionnaires as their primary outcome measure. Clinical interviews are of course more labour-intensive, but the diagnostic accuracy might be higher (Ferrari et al., 2013), especially in non-Western cultural contexts. If the planned outcome measure for the psychological trial is a clinical interview, it is recommended to integrate a culture-sensitive interview, such as the *Cultural Formulation Interview* in DSM-5 (American Psychiatric Association, 2013). Training interviewers in culture-sensitive assessments is important, in order to avoid misdiagnosis. And it is relevant to report on interviewer training and interrater reliability with regard to cultural competence.

Do you agree with the description?

- Strongly agree
- Somewhat agree
- Somewhat disagree
- Strongly disagree

61 Criterion 11. Clinical interviews

Criterion 11. Clinical interviews

Most trials use self-report questionnaires as their primary outcome measure. Clinical interviews are of course more labour-intensive, but the diagnostic accuracy might be higher (Ferrari et al., 2013), especially in non-Western cultural contexts. If the planned outcome measure for the psychological trial is a clinical interview, it is recommended to integrate a culture-sensitive interview, such as the *Cultural Formulation Interview* in DSM-5 (American Psychiatric Association, 2013). Training interviewers in culture-sensitive assessments is important, in order to avoid misdiagnosis. And it is relevant to report on interviewer training and interrater reliability with regard to cultural competence.

Do you have any comments on the criterion and the text?

62 Criterion 11. Clinical interviews

Criterion 11. Clinical interviews

Please rate the relevance of this criterion

- Totally relevant
- Somewhat relevant
- Hardly relevant
- Not at all relevant

63 Criterion 11. Clinical interviews

Criterion 11. Clinical interviews

Please rate the applicability of this criterion

- Totally applicable
- Somewhat applicable
- Hardly applicable
- Not at all applicable

64 Other reporting criteria

Would you like to suggest other reporting criteria?

Please provide a short description.

65 Other experts

Would you like to suggest experts for this review?

Please provide a valid e-mail address.

66 Thanks

Many thanks for your valuable contribution. We will contact you for the next round of feedback in 3-4 weeks from now.

Kind regards

Eva Heim (e.heim@psychologie.uzh.ch**)**

References

Abi Ramia, J., Harper Shehadeh, M., Kheir, W., Zoghbi, E., Watts, S., Heim, E., & El Chammay, R. (2018). Community cognitive interviewing to inform local adaptations of an e-mental health intervention in Lebanon. *Global Mental Health, 5*, e39. https://doi.org/10.1017/gmh.2018.29

American Psychiatric Association. (2013). *Diagnostic and statistical manual of mental disorders (5th ed.)*. American Psychiatric Publishing.

Applied Mental Health Research (AMHR) Group at Johns Hopkins University. (2013). *The DIME Program Research Model: Design, Implementation, Monitoring and Evaluation*. https://www.jhsph.edu/research/centers-and-institutes/global-mental-health/resource-materials/design-implementation-monitoring-and-evaluation-dime/

Ashworth, M., Shepherd, M., Christey, J., Matthews, V., Wright, K., Parmentier, H., Robinson, S., & Godfrey, E. (2004). A client-generated psychometric instrument: The development of 'PSYCHLOPS'. *Counselling and Psychotherapy Research, 4*(2), 27-31. https://doi.org/10.1080/14733140412331383913

Bernal, G., Bonilla, J., & Bellido, C. (1995). Ecological validity and cultural sensitivity for outcome research: issues for the cultural adaptation and development of psychosocial treatments with Hispanics. *Journal of Abnormal Child Psychology, 23*, 67–82.

Bernal, G., & Sáez-Santiago, E. (2006). Culturally centered psychosocial interventions. *Journal of Community Psychology, 34*(2), 121-132. https://doi.org/10.1002/jcop.20096

Betancourt, H., & López, S. R. (1993). The study of culture, ethnicity, and race in American psychology. *American Psychologist, 48*(6), 629-637. https://doi.org/10.1037/0003-066X.48.6.629

Bhui, K., & Bhugra, D. (2002). Explanatory models for mental distress: Implications for clinical practice and research [10.1192/bjp.181.1.6]. *The British Journal of Psychiatry, 181*(1), 6.

Burchert, S., Alkneme, M. S., Bird, M., Carswell, K., Cuijpers, P., Hansen, P., Heim, M., Harper Shehadeh, M., Sijbrandij, M., Van't Hof, E., & Knaevelsrud, C. (2019). User-centered app adaptation of a low-intensity e-mental health intervention for Syrian refugees. *Frontiers in Psychiatry, 9*, 663.

Byrne, B. M., Shavelson, R. J., & Muthén, B. (1989). Testing for the equivalence of factor covariance and mean structures: The issue of partial measurement invariance. *Psychological Bulletin, 105*(3), 456-466.

Carswell, K., Harper-Shehadeh, M., Watts, S., van't Hof, E., Abi Ramia, J., Heim, E., Wenger, A., & van Ommeren, M. (2018). Step-by-Step: a new WHO digital mental health intervention for depression. *mHealth, 4*(8). http://mhealth.amegroups.com/article/view/20772

Castro, F. G., Barrera, M., Jr., & Holleran Steiker, L. K. (2010). Issues and challenges in the design of culturally adapted evidence-based interventions. *Annual Review of Clinical Psychology, 6*, 213-239. https://doi.org/10.1146/annurev-clinpsy-033109-132032

Chambers, D. A., Glasgow, R. E., & Stange, K. C. (2013). The dynamic sustainability framework: addressing the paradox of sustainment amid ongoing change. *Implementation Science, 8*(1), 117. https://doi.org/10.1186/1748-5908-8-117

Chambers, D. A., & Norton, W. E. (2016). The Adaptome: Advancing the science of intervention adaptation. *Am J Prev Med, 51*(4 Suppl 2), S124-131. https://doi.org/10.1016/j.amepre.2016.05.011

Chorpita, B. F., & Daleiden, E. L. (2009). Mapping evidence-based treatments for children and adolescents: application of the distillation and matching model to 615 treatments from 322 randomized trials. *Journal of Consulting and Clinical Psychology, 77*(3), 566-579. https://doi.org/10.1037/a0014565

Chowdhary, N., Jotheeswaran, A. T., Nadkarni, A., Hollon, S. D., King, M., Jordans, M. J., Rahman, A., Verdeli, H., Araya, R., & Patel, V. (2014). The methods and outcomes of cultural adaptations of psychological treatments for depressive disorders: A systematic review. *Psychol Med, 44*(6), 1131-1146. https://doi.org/10.1017/s0033291713001785

Chu, J., & Leino, A. (2017). Advancement in the maturing science of cultural adaptations of evidence-based interventions. *Journal of Consulting and Clinical Psychology, 85*(1), 45-57. https://doi.org/10.1037/ccp0000145

Cork, C., Kaiser, B. N., & White, R. G. (2019). The integration of idioms of distress into mental health assessments and interventions: a systematic review. *Global Mental Health, 6*, e7, Article e7. https://doi.org/10.1017/gmh.2019.5

Cuijpers, P., Reijnders, M., & Huibers, M. J. H. (2019). The role of common ractors in psychotherapy outcomes. *Annual Review of Clinical Psychology, 15*, 207-231. https://doi.org/10.1146/annurev-clinpsy-050718-095424

Czachowski, S., Seed, P., Schofield, P., & Ashworth, M. (2011). Measuring psychological change during cognitive behaviour therapy in primary care: a polish study using 'PSYCLOPS' (Psychological Outcome Profiles). *PLoS One, 6*(12), e27378. https://doi.org/10.1371/journal.pone.0027378

Dawson, K. S., Bryant, R. A., Harper, M., Kuowei Tay, A., Rahman, A., Schafer, A., & van Ommeren, M. (2015). Problem Management Plus (PM+): a WHO transdiagnostic psychological intervention for common mental health problems. *World Psychiatry, 14*(3), 354-357. https://doi.org/10.1002/wps.20255

Epping-Jordan, J. E., Harris, R., Brown, F. L., Carswell, K., Foley, C., García-Moreno, C., Kogan, C., & van Ommeren, M. (2016). Self-Help Plus (SH+): A new WHO stress management package. *World Psychiatry, 15*(3), 295-296. https://doi.org/10.1002/wps.20355

Ertl, V., Pfeiffer, A., Schauer, E., Elbert, T., & Neuner, F. (2011). Community-implemented trauma therapy for former child soldiers in Northern Uganda: a randomized controlled trial. *JAMA, 306*(5), 503-512. https://doi.org/10.1001/jama.2011.1060

Ferrari, A., Somerville, A., Baxter, A., Norman, R., Patten, S., Vos, T., & Whiteford, H. (2013). Global variation in the prevalence and incidence of major depressive disorder: A systematic review of the epidemiological literature. *Psychological Medicine, 43*(3), 471-481. https://doi.org/10.1017/s0033291712001511

Ferster, C. B. (1973). A functional analysis of depression. *American Psychologist, 28*(10), 857-870. https://doi.org/10.1037/h0035605

Fischer, R., & Schwartz, S. (2011). Whence differences in value priorities? Individual, cultural, or artifactual sources. *Journal of Cross-Cultural Psychology, 42*(7), 1127-1144. https://doi.org/10.1177/0022022110381429

Garabiles, M. R., Harper Shehadeh, M., & Hall, B. J. (2019). Cultural adaptation of a scalable World Health Organization e-mental health program for overseas Filipino workers [Original Paper]. *JMIR Formative Research, 3*(1), e11600. https://doi.org/10.2196/11600

Gautham, M., Singh, R., Weiss, H., Brugha, R., Patel, V., Desai, N. G., Nandan, D., Kielmann, K., & Grosskurth, H. (2008). Socio-cultural, psychosexual and biomedical factors associated with genital symptoms experienced by men in rural India. *Tropical Medicine & International Health, 13*(3), 384-395. https://doi.org/10.1111/j.1365-3156.2008.02013.x

Gearing, R. E., Schwalbe, C. S., MacKenzie, M. J., Brewer, K. B., Ibrahim, R. W., Olimat, H. S., Al-Makhamreh, S. S., Mian, I., & Al-Krenawi, A. (2012). Adaptation and translation of mental health interventions in Middle Eastern Arab countries: A systematic review of barriers to and strategies for effective treatment implementation. *International Journal of Social Psychiatry, 59*(7), 671-681. https://doi.org/10.1177/0020764012452349

Greene, M. C., Jordans, M. J. D., Kohrt, B. A., Ventevogel, P., Kirmayer, L. J., Hassan, G., Chiumento, A., van Ommeren, M., & Tol, W. A. (2017). Addressing culture and context in humanitarian response: preparing desk reviews to inform mental health and psychosocial support [journal article]. *Conflict and Health, 11*(1), 21. https://doi.org/10.1186/s13031-017-0123-z

Groleau, D., Young, A., & Kirmayer, L. J. (2006). The McGill Illness Narrative Interview (MINI): An interview schedule to elicit meanings and modes of reasoning related to illness experience. *Transcultural Psychiatry, 43*(4), 671-691. https://doi.org/10.1177/1363461506070796

Hall, G. C. N., Ibaraki, A. Y., Huang, E. R., Marti, C. N., & Stice, E. (2016). A meta-analysis of cultural adaptations of psychological interventions. *Behavior Therapy, 47*(6), 993-1014. https://doi.org/10.1016/j.beth.2016.09.005

Haroz, E. E., Ritchey, M., Bass, J. K., Kohrt, B. A., Augustinavicius, J., Michalopoulos, L., Burkey, M. D., & Bolton, P. (2017). How is depression experienced around the world? A systematic review of qualitative literature. *Social Science & Medicine, 183*, 151-162. https://doi.org/10.1016/j.socscimed.2016.12.030

Harper Shehadeh, M., Heim, E., Chowdhary, N., Maercker, A., & Albanese, E. (2016). Cultural adaptation of minimally guided interventions for common mental disorders: A systematic review and meta-analysis [Original Paper]. *JMIR Mental Health, 3*(3), e44. https://doi.org/10.2196/mental.5776

Héðinsson, H., Kristjánsdóttir, H., Ólason, D. Þ., & Sigurðsson, J. F. (2013). A validation and replication study of the patient-generated measure PSYCHLOPS on an Icelandic clinical population. *European Journal of Psychological Assessment, 29*(2), 89-95. https://doi.org/10.1027/1015-5759/a000136

Heim, E., Burchert, S., Shala, M., Kaufmann, M., Cerga-Pashoja, A., Morina, N., Schaub, M. P., Knaevelsrud, C., & Maercker, A. (2020). Effect of cultural adaptation of a smartphone-based self-help programme on its acceptability and efficacy: study protocol for a randomized controlled trial. *PsychArchives*. https://doi.org/http://dx.doi.org/10.23668/psycharchives.3152.

Heim, E., & Kohrt, B. A. (2019). Cultural adaptation of scalable psychological interventions: a new conceptual framework. *Clinical Psychology in Europe, 1*(4), e37679. https://doi.org/10.32872/cpe.v1i4.37679

Hinton, D. E., Rivera, E. I., Hofmann, S. G., Barlow, D. H., & Otto, M. W. (2012). Adapting CBT for traumatized refugees and ethnic minority patients: examples from culturally adapted CBT (CA-CBT). *Transcultural Psychiatry, 49*(2), 340-365. https://doi.org/10.1177/1363461512441595

Hinton, D. E., Seponski, D. M., Khann, S., Armes, S. E., Lahar, C. J., Kao, S., & Schunert, T. (2019). Culturally sensitive assessment of anxious-depressive distress in the Cambodian population: Avoiding category truncation. *Transcultural Psychiatry, 56*(4), 643-666. https://doi.org/10.1177/1363461519851609

Hwang, W.-C. (2006). The psychotherapy adaptation and modification framework: Application to Asian Americans. *American Psychologist, 61*(7), 702-715. https://doi.org/10.1037/0003-066X.61.7.702

Kaiser, B. N., & Jo Weaver, L. (2019). Culture-bound syndromes, idioms of distress, and cultural concepts of distress: New directions for an old concept in psychological anthropology. *Transcultural Psychiatry, 56*(4), 589-598. https://doi.org/10.1177/1363461519862708

Keys, H. M., Kaiser, B. N., Kohrt, B. A., Khoury, N. M., & Brewster, A. R. (2012). Idioms of distress, ethnopsychology, and the clinical encounter in Haiti's Central Plateau. *Social Science & Medicine, 75*(3), 555-564. https://doi.org/10.1016/j.socscimed.2012.03.040

Knaevelsrud, C., Brand, J., Lange, A., Ruwaard, J., & Wagner, B. (2015). Web-based psychotherapy for posttraumatic stress disorder in war-traumatized arab patients: randomized controlled trial. *J Med Internet Res, 17*(3), e71. https://doi.org/10.2196/jmir.3582

Kohrt, B. A., Rasmussen, A., Kaiser, B. N., Haroz, E. E., Maharjan, S. M., Mutamba, B. B., de Jong, J. T., & Hinton, D. E. (2014). Cultural concepts of distress and psychiatric disorders: Literature review and research recommendations for global mental health epidemiology. *International Journal of Epidemiology, 43*(2), 365-406. https://doi.org/10.1093/ije/dyt227

Lehman, D. R., Chiu, C.-y., & Schaller, M. (2004). Psychology and culture. *Annual Review of Psychology, 55*(1), 689-714. https://doi.org/10.1146/annurev.psych.55.090902.141927

Lewinsohn, P. M. (1974). A behavioral approach to depression. In R. J. Freidman & M. Katz (Eds.), *The psychology of depression: contemporary theory and research* (pp. 157-178). Wiley.

Lloyd, K. R., Jacob, K. S., Patel, V., St Louis, L., Bhugra, D., & Mann, A. H. (1998). The development of the Short Explanatory Model Interview (SEMI) and its use among primary-care attenders with common mental disorders.

Psychological Medicine, 28(5), 1231-1237.

Malterud, K. (2001). Qualitative research: standards, challenges, and guidelines. *The Lancet, 358*(9280), 483-488. https://doi.org/https://doi.org/10.1016/S0140-6736(01)05627-6

Milfont, T. L., & Fischer, R. (2010). Testing measurement invariance across groups: Applications in cross-cultural research [measurement invariance; cross-cultural research; confirmatory factor analysis; LISREL]. *International Journal of Psychological Research, 3*(1), 111-121. http://mvint.usbmed.edu.co:8002/ojs/index.php/web/article/view/465

Min, S. K., & Suh, S.-y. (2010). The anger syndrome hwa-byung and its comorbidity. *Journal of Affective Disorders, 124*(1), 211-214. https://doi.org/https://doi.org/10.1016/j.jad.2009.10.011

Mummah, S. A., Robinson, T. N., King, A. C., Gardner, C. D., & Sutton, S. (2016). IDEAS (Integrate, Design, Assess, and Share): A Framework and Toolkit of Strategies for the Development of More Effective Digital Interventions to Change Health Behavior [Viewpoint]. *Journal of Medical Internet Research, 18*(12), e317. https://doi.org/10.2196/jmir.5927

Murray, L. K., Dorsey, S., Haroz, E., Lee, C., Alsiary, M. M., Haydary, A., Weiss, W. M., & Bolton, P. (2014). A common elements treatment approach for adult mental health problems in low- and middle-income countries. *Cogn Behav Pract, 21*(2), 111-123. https://doi.org/10.1016/j.cbpra.2013.06.005

Naslund, J. A., Aschbrenner, K. A., Araya, R., Marsch, L. A., Unutzer, J., Patel, V., & Bartels, S. J. (2017). Digital technology for treating and preventing mental disorders in low-income and middle-income countries: a narrative review of the literature. *Lancet Psychiatry, 4*(6), 486-500. https://doi.org/10.1016/s2215-0366(17)30096-2

Newby, J. M., Mackenzie, A., Williams, A. D., McIntyre, K., Watts, S., Wong, N., & Andrews, G. (2015). Internet cognitive behavioural therapy for mixed anxiety and depression: a randomized controlled trial and evidence of effectiveness in primary care. *Psychol Med, 43*(12), 2635-2648. https://doi.org/10.1017/s0033291713000111

Nichter, M. (1981). Idioms of distress: Alternatives in the expression of psychosocial distress: A case study from South India. *Culture, Medicine, and Psychiatry, 5*(4), 379-408.

Nichter, M. (2010). Idioms of distress revisited. *Culture, Medicine, and Psychiatry, 34*(2), 401-416. https://doi.org/10.1007/s11013-010-9179-6

Patel, V., Gwanzura, F., Simunyu, E., Lloyd, K., & Mann, A. (1995). The phenomenology and explanatory models of common mental disorder: a study in primary care in Harare, Zimbabwe. *Psychological Medicine, 25*(6), 1191-1199. https://doi.org/10.1017/S003329170003316X

Perera, C., Salamanca-Sanabria, A., Caballero-Bernal, J., Feldman, L., Hansen, M., Bird, M., Hansen, P., Dinesen, C., Wiedemann, N., & Vallières, F. (2020). No implementation without cultural adaptation: a process for culturally adapting low-intensity psychological interventions in humanitarian settings. *Conflict and Health, 14*(1), 46. https://doi.org/10.1186/s13031-020-00290-0

Ramaiya, M. K., Fiorillo, D., Regmi, U., Robins, C. J., & Kohrt, B. A. (2017). A cultural adaptation of Dialectical Behavior Therapy in Nepal. *Cogn Behav Pract, 24*(4), 428-444. https://doi.org/10.1016/j.cbpra.2016.12.005

Rasmussen, A., Keatley, E., & Joscelyne, A. (2014). Posttraumatic stress in emergency settings outside North America and Europe: a review of the emic literature. *Social Science & Medicine, 109*, 44-54. https://doi.org/10.1016/j.socscimed.2014.03.015

Rathod, S., Phiri, P., & Naeem, F. (2019). An evidence-based framework to culturally adapt cognitive behaviour therapy. *The Cognitive Behaviour Therapist, 12*, e10, Article e10. https://doi.org/10.1017/S1754470X18000247

Rechsteiner, K., Maercker, A., Heim, E., & Meili, I. (2020). Metaphors for trauma: a cross-cultural qualitative comparison in Brazil, India, Poland, and Switzerland. *Journal of Traumatic Stress, n/a*(n/a). https://doi.org/10.1002/jts.22533

Resnicow, K., Baranowski, T., Ahluwalia, J. S., & Braithwaite, R. L. (1999). Cultural sensitivity in public health: defined and demystified. *Ethnicity & Disease, 9*(1), 10-21.

Rüdell, K., Bhui, K., & Priebe, S. (2009). Concept, development and application of a new mixed method assessment of cultural variations in illness perceptions: Barts explanatory model inventory. *Journal of Health Psychology, 14*(2), 336-347. https://doi.org/10.1177/1359105308100218

Salamanca-Sanabria, A., Richards, D., & Timulak, L. (2019). Adapting an internet-delivered intervention for depression for a Colombian college student population: An illustration of an integrative empirical approach. *Internet Interventions, 15*, 76-86. https://doi.org/10.1016/j.invent.2018.11.005

Sangraula, M., Van't Hof, E., Luitel, N. P., Turner, E. L., Marahatta, K., Nakao, J. H., van Ommeren, M., Jordans, M. J. D., & Kohrt, B. A. (2018). Protocol for a feasibility study of group-based focused psychosocial support to improve the psychosocial well-being and functioning of adults affected by humanitarian crises in Nepal: Group Problem Management Plus (PM+). *Pilot and Feasibility Studies, 4*, 126-126. https://doi.org/10.1186/s40814-018-0315-3

Shala, M., Morina, N., Burchert, S., Cerga-Pashoja, A., Knaevelsrud, C., Maercker, A., & Heim, E. (2020). Cultural adaptation of Hap-pas-Hapi, an internet and mobile-based intervention for the treatment of psychological distress among Albanian migrants in Switzerland and Germany. *Internet Interventions, 21*, 100339. https://doi.org/https://doi.org/10.1016/j.invent.2020.100339

Shala, M., Morina, N., Burchert, S., Cerga-Pashoja, A., Knaevelsrud, C., Maercker, A., & Heim, E. (accepted). Cultural adaptation of Hap-pas-Hapi, an internet and mobile-based intervention for the treatment of depression among Albanian immigrants in Switzerland and Germany. *Internet Interventions*.

Shala, M., Morina, N., Salis Gross, C., Maercker, A., & Heim, E. (2020). A point in the heart: concepts of emotional distress among Albanian-speaking immigrants in Switzerland. *Culture, Medicine, and Psychiatry, 40*, 1–34. https://doi.org/10.1007/s11013-019-09638-5

Singla, D. R., Kohrt, B. A., Murray, L. K., Anand, A., Chorpita, B. F., & Patel, V. (2017). Psychological treatments for the world: lessons from low- and middle-income countries. *Annual Review of Clinical Psychology, 13*(1), 149-181. https://doi.org/10.1146/annurev-clinpsy-032816-045217

Sue, D. W., & Sue, D. (2015). *Counseling the culturally diverse: theory and practice*. Wiley.

Tong, A., Craig, J., & Sainsbury, P. (2007). Consolidated criteria for reporting qualitative research (COREQ): a 32-item checklist for interviews and focus groups. *International Journal for Quality in Health Care, 19*(6), 349-357. https://doi.org/10.1093/intqhc/mzm042

Van Ommeren, M., Sharma, B., Thapa, S., Makaju, R., Prasain, D., Bhattarai, R., & de Jong, J. (1999). Preparing instruments for transcultural research: use of the translation monitoring form with Nepali-speaking Bhutanese refugees. *Transcultural Psychiatry, 36*(3), 285-301. https://doi.org/10.1177/136346159903600304

Vandenberg, R. J., & Lance, C. E. (2000). A review and synthesis of the measurement invariance literature: Suggestions, practices, and recommendations for organizational research. *Organizational Research Methods, 3*(1), 4-70. https://doi.org/10.1177/109442810031002

Veale, D. (2008). Behavioural activation for depression. *Advances in Psychiatric Treatment, 14*(1), 29-36. https://doi.org/10.1192/apt.bp.107.004051

Verdeli, H., Clougherty, K., Bolton, P., Speelman, L., Lincoln, N., Bass, J., Neugebauer, R., & Weissman, M. (2003). Adapting group interpersonal psychotherapy for a developing country: experience in rural Uganda. *World Psychiatry, 2*, 114 - 120.

Wampold, B. (2007). Psychotherapy: the humanistic (and effective) treatment. *American Psychologist, 62*(8), 855-873. https://doi.org/10.1037/0003-066x.62.8.857

Wild, D., Grove, A., Martin, M., Eremenco, S., McElroy, S., Verjee-Lorenz, A., & Erikson, P. (2005). Principles of good practice for the translation and cultural adaptation process for patient-reported outcomes (PRO) measures. *Value in Health, 8*(2), 95–104. https://doi.org/10.1111/j.1524-4733.2005.04054.x

Willis, G. B. (2004). *Cognitive interviewing: A tool for improving questionnaire design*. Sage Publications.

Wingood, G. M., & DiClemente, R. J. (2008). The ADAPT-ITT model: A novel method of adapting evidence-based HIV interventions. *JAIDS Journal of Acquired Immune Deficiency Syndromes, 47*. https://journals.lww.com/jaids/Fulltext/2008/03011/The_ADAPT_ITT_Model__A_Novel_Method_of_Adapting.8.aspx

Yang, L. H., Thornicroft, G., Alvarado, R., Vega, E., & Link, B. G. (2014). Recent advances in cross-cultural measurement in psychiatric epidemiology: Utilizing 'what matters most' to identify culture-specific aspects of stigma. *International Journal of Epidemiology, 43*(2), 494-510. https://doi.org/10.1093/ije/dyu039

67 Final page
