# Supplementary material for: Reporting Cultural Adaptation in Psychological Trials – The RECAPT criteria
Source: Clin Psychol Eur. 2021 Nov 23;3(Spec Issue):e6351. doi: 10.32872/cpe.6351 (PMC9670826; doi:10.32872/cpe.6351)
Supplement: Supplement 2 [file cpe-03-6351-s02.pdf]

Reporting Cultural Adaptation in Psychological Trials - The RECAPT criteria
Eva Heim, Ricarda Mewes, et al., 2021
Manuscript published in Clinical Psychology in Europe, doi:https://doi.org/10.32872/cpe.6351

Template for documenting cultural adaptations of psychological interventions

Low quality of reporting: 4 or fewer
Moderate quality of reporting: 5-8 [Minimum criteria]
High quality of reporting: 9 or more [Most criteria]

A) Set-up

Criterion 1: Definition of the target population

(Text in italics highlights the sections completed by researchers and provides examples)

Category	Description	Results
Target group	Description of elements that are potentially relevant for cultural adaptation, e.g. language, religion, migration status, age	*Adult Afghan and Syrian refugees in Germany; middle-age adults (25-65); Arabic speaking with limited German proficiency; resettlement in Germany between 1-5 years prior to the adaptation process*

Criterion 2: Team and roles

This table presents relevant information related to the researchers' backgrounds. Columns can be re-named if necessary.

(Text in italics highlights the sections completed by researchers and provides examples)

Team member	Gender	Disciplinary background	Level / experience /other relevant information	Cultural characteristics	Role
Researcher 1	*female*	*Medical anthropology*	*Post-doctoral researchers (4 years after PhD)*	*4 years of experience living in community comparable to target population*	*Responsible for cultural adaptatation, i.e., literature review, implementation of focus groups, documentation, monitoring*
Researcher 2	*female*	*Clinical psychologist*	*Professor (12 years after PhD)*		*Supervisor, discussant*
Researcher 3	*male*	*Clinical psychologist*	*Professor (20 years after PhD),*	*native-speaker*	*Expert, discussant*
Mental health advocate/ service user	*female*	*Peer specialist provider*	*Bachelors degree*	*Resident of community comparable to target population*	*Expert, discussant*

B) Formative research

**Criterion 5: Formative research methods**<br>*(Text in italics highlights the sections completed by researchers and provides examples)*			
**Category**	**Suggested sources (not exhaustive)**	**Suggested items to be reported**	**Results**
Literature review	Desk review (Greene et al., 2017)	• Searched databases<br>• Search terms<br>• If applicable, inclusion / exclusion criteria<br>• Identified references	
Qualitative methods	For reporting qualitative methods:<br>• Consolidated criteria for reporting qualitative research (COREQ, Tong et al., 2007)<br><br>For assessing cultural concepts of distress<br>• *Cultural Formulation Interview* in DSM-5 (American Psychiatric Association, 2013),<br>• *Short Explanatory Model Interview* (SEMI, Lloyd et al., 1998),<br>• *Barts Explanatory Model Inventory* (BEMI, Rüdell et al., 2009)<br>• *McGill Illness Narrative Interview* (MINI, Groleau et al., 2006).<br><br>For data collection on intervention adaptation:<br>• Cognitive interviewing (Willis, 2004)<br>• Cultural Relevance Questionnaire (CRQ, Salamanca-Sanabria et al., 2019)	• Participant selection (i.e., selection, method of approach, sample size, reasons for refusing);<br>• Setting for data collection (e.g., home, clinic);<br>• Method of data collection (i.e., interview guide, recording, duration)<br>• Analysis methods (i.e., how themes were derived from the data)	*We sought to provide insights into beliefs about causes of posttraumatic stress disorder (PTSD) held by Sub-Saharan African asylum seekers living in Germany. To this aim, we used a quantitative and qualitative methodological triangulation strategy based on a vignette describing symptoms of PTSD. In the qualitative part of the study, asylum seekers reviewed the results of the quantitative study part within eight focus group discussions (n = 26), sampled from groups of the three main countries of origin. Focus group discussions were moderated using key questions from the SEMI. The discussions took part in prepared interview rooms throughout different cities in Germany. The average duration of the focus group discussions was 1 h 30min. Focus group discussions were recorded, transcribed, and analyzed using Interpretative Phenomenological Analysis (IPA). The software MAXQDA© version 12 was used to organize and manage data analysis. Within the focus group discussions, six attribution categories of participants' causal beliefs were identified: (a) traumatic life experiences, (b) psychological causes, (c) social causes, (d) post-migration stressors, (e) religious causes, and (f) supernatural causes (Grupp et al., 2018).*
Quantitative methods	For reporting quantitative methods:<br>STROBE Statement (https://www.strobe-statement.org/index.php?id=strobe-home)	• Study aim and hypotheses<br>• Participants (i.e., selection, method of approach, sample size, reasons for refusing);<br>• Study design (e.g., experimental, cross-secional, longitudinal);<br>• Method of data collection (i.e., interviews, questionnaires)<br>• Analysis methods	*Objective: We aimed to provide insights into help-seeking intentions and lay beliefs about cures for PTSD held by asylum seekers from Sub-Saharan Africa living in Germany. We hypothesized that Sub-Saharan African asylum seekers would express a higher intention to seek religious and medical treatment and a lower intention to seek psychological treatment than German participants without a migration background. Methods: To address this objective, we used a quantitative and qualitative methodological triangulation strategy based on a vignette describing symptoms of PTSD. In the quantitative part of the study, asylum seekers (n=119), predominantly from Eritrea (n=41), Somalia (n= 36), and Cameroon (n = 25), and a German comparison sample without a migration background (n=120) completed the General Help-Seeking Questionnaire (GHSQ).*

**Criterion 5: Formative research methods**<br>*(Text in italics highlights the sections completed by researchers and provides examples)*			
**Category**	**Suggested sources (not exhaustive)**	**Suggested items to be reported**	**Results**
			Results: Asylum seekers showed a high intention to seek religious, medical, and psychological treatment for symptoms of PTSD. However, asylum seekers indicated a higher preference to seek help from religious authorities and general practitioners, as well as a lower preference to enlist psychological and traditional help sources than Germans without a migration background (Grupp et al., 2019).

**Criterion 6: Target symptoms, syndromes, needs, and context**<br>*(Text in italics highlights the sections completed by researchers and provides examples)*			
**Category**	**Description**	**Results**	**Source**
Idioms of distress, specific target symptoms	Socially acceptable terms for expressing distress, culturally salient symptoms	• *Thinking too much*<br>• *Point in the heart*<br>• *Anger / aggression*<br>• *Withdrawal*<br>• *Pain*	• *Literature review (cite references)*<br>• *Focus groups*<br>• *Key informant interviews*
Explanatory and ethnopsychological models	General assumptions about human suffering, disorder-specific assumptions / beliefs; models relating suffering to concepts of the self, body, emotions, etc.	• *Human suffering is caused by god or fate*	• *Literature review (cite references)*<br>• *Focus groups*<br>• *Key informant interviews*
Cultural concepts of distress	Syndromes described for a particular target group	• *khyâl attacks*	• *Literature review (cite references)*<br>• *Focus groups*<br>• *Key informant interviews*
Beliefs about the course of the disorder and help-seeking behaviour	General assumptions about and healing, coping, and help-seeking, specific strategies applied before	• *Fatalism: Suffering is part of human life and has to be endured with patience*	• *Literature review (cite references)*<br>• *Focus groups*<br>• *Key informant interviews*
Mental health related stigma	• In community samples: Knowledge, attitude and behaviours related to people with mental disorders<br>• In clinical samples: Experienced and anticipated negative attitudes and behaviours in the family and community	• *People with substance abuse disorders are to be blamed*	• *Literature review (cite references)*

**Criterion 6: Target symptoms, syndromes, needs, and context**<br>*(Text in italics highlights the sections completed by researchers and provides examples)*			
**Category**	**Description**	**Results**	**Source**
Specific needs and other relevant contextual information	Differential exposure to social determinants of mental health, other contextual information, access to health systems, and mental health resources	• *Ongoing armed conflict*	• *Key informant interviews*

C) Intervention adaptation

Criterion 7: Specific treatment elements

Criterion 8: Unspecific elements and therapeutic techniques

(Text in italics highlights the sections completed by researchers and provides examples)

**Decision-Nr.**	**Mechanisms of action including treatment elements, techniques, delivery, surface**	**Original intervention**	**Cultural Processes related to mechanism of action**	**Cultural / contextual adaptation**	**Evidence base**<br><br>e.g., literature review, focus groups, qualitative interview	**Quality of evidence**<br><br>Strong<br>Moderate<br>Weak	**Suggestions from research team**				**State of decision**	
							**Researcher 1**	**Researcher 2**	**Researcher 3**	**Researcher 4**	**pending**	**made**
1	*Specific factor*	-	*Cultural model of tension generated by inability to manage problems*	*Problem management added to address post-migration stressors*							x	
2	*Specific factor*	*Emotion regulation*	*Cultural model of generating calmness in the heart-mind to respond to strong emotions*	*Use of religious rituals as a strategy to regulate strong emotions*							x	
3	*Unspecific factor*	*Psychoeducation*	*Cultural model of strong emotions manifested as somatic complaints (e.g., headaches, heaviness in the body, numbness or tingling)*	*Information on somatic symtpoms included*								x

Criterion 9: Surface adaptations

(Text in italics highlights the sections completed by researchers and provides examples)

**Decision-Nr.**	**Treatment elements, techniques, delivery, surface**	**Original intervention**	**Cultural / contextual adaptation**	**Evidence base**<br><br>e.g., literature review, focus groups, qualitative interview	**Quality of evidence**<br><br>Strong<br>Moderate<br>Weak	**Suggestions from research team**				**State of decision**	
						**Researcher 1**	**Researcher 2**	**Researcher 3**	**Researcher 4**	**pending**	**made**
4	*Treatment delivery*	*Twelve sessions*	*Five sessions*							x	
5	*Surface*	*Written text on relaxation*	*Illustrations instead of written text*								x

D) Measuring outcomes

Criterion 10: Questionnaires and clinical interviews

Criterion 11: Implementation measures

(Text in italics highlights the sections completed by researchers and provides examples)

**Category**	**Instruments used for outcome assessments**	**Translation / validation / adaptation**	**Sources**
Clinical Interviews	• *Cultural Formulation Interview in DSM-5*	• *Translated and back-translated by study team*	American Psychiatric Association (2013)
Questionnaires	*Adaptation guidelines:*<br>• *Transcultural Translation and Adaptation Monitoring Form (van Ommeren et al., 1999)*<br><br>*Examples of instruments*<br>• *Generalized Anxiety Disorder – 7 Questionnaire (GAD-7)*<br>• *Patient Health Questionnaire – 9 (PHQ-9)*	• *Validated for study population*	van Ommeren et al. (1999)<br><br>Kroenke and Spitzer (2002)<br>Spitzer et al. (2006)<br>Sawaya et al. (2016)
Implementation Measures	*Fidelity to intervention*<br>• *Problem Management Plus (PM+) treatment fidelity measure*<br><br>*Competency of persons delivering intervention (nonspecific factors)*<br>• *ENhancing Assessment of Common Therapeutic Factors (ENACT)*<br><br>*Competency of persons delivering intervention (treatment specific factors)*<br>• *EQUIP Problem Management Plus (PM+) Competency scale*	• *PM+ fidelity scale modified to include cultural adaptations for Nepal*<br><br>• *ENACT competency rating scale developed in Nepal and culturally modified for use in other low- and middle-income country settings*<br><br>• *PM+ competency scale adapted into Tigrinya and for use in refugee camps in Ethiopia*	Sangraula et al. (2020)<br>Kohrt et al. (2015)<br>Pedersen et al. (in press)

References

American Psychiatric Association. (2013). *Diagnostic and statistical manual of mental disorders (5th ed.)*. American Psychiatric Publishing.

Greene, M. C., Jordans, M. J. D., Kohrt, B. A., Ventevogel, P., Kirmayer, L. J., Hassan, G., Chiumento, A., van Ommeren, M., & Tol, W. A. (2017, November 15). Addressing culture and context in humanitarian response: preparing desk reviews to inform mental health and psychosocial support [journal article]. *Conflict and Health, 11*(1), 21. https://doi.org/10.1186/s13031-017-0123-z

Groleau, D., Young, A., & Kirmayer, L. J. (2006, Dec). The McGill Illness Narrative Interview (MINI): An interview schedule to elicit meanings and modes of reasoning related to illness experience. *Transcultural Psychiatry, 43*(4), 671-691. https://doi.org/10.1177/1363461506070796

Grupp, F., Moro, M. R., Nater, U. M., Skandrani, S., & Mewes, R. (2019, 2019/12/31). 'Only God can promise healing.': help-seeking intentions and lay beliefs about cures for post-traumatic stress disorder among Sub-Saharan African asylum seekers in Germany. *European Journal of Psychotraumatology, 10*(1), 1684225. https://doi.org/10.1080/20008198.2019.1684225

Grupp, F., Moro, M. R., Nater, U. M., Skandrani, S. M., & Mewes, R. (2018, 2018-November-26). "It's that route that makes us sick": exploring lay beliefs about causes of post-traumatic stress disorder among Sub-saharan African asylum seekers in Germany [Original Research]. *Frontiers in Psychiatry, 9*(628). https://doi.org/10.3389/fpsyt.2018.00628

Kohrt, B. A., Jordans, M. J., Rai, S., Shrestha, P., Luitel, N. P., Ramaiya, M. K., Singla, D. R., & Patel, V. (2015, Jun). Therapist competence in global mental health: Development of the ENhancing Assessment of Common Therapeutic factors (ENACT) rating scale. *Behaviour Research and Therapy, 69*, 11-21. https://doi.org/10.1016/j.brat.2015.03.009

Kroenke, K., & Spitzer, R. (2002). The PHQ-9: a new depression diagnostic and severity measure. *Psychiatric Annals, 32*, 91 - 97.

Lloyd, K. R., Jacob, K. S., Patel, V., St Louis, L., Bhugra, D., & Mann, A. H. (1998, Sep). The development of the Short Explanatory Model Interview (SEMI) and its use among primary-care attenders with common mental disorders. *Psychological Medicine, 28*(5), 1231-1237.

Pedersen, G., Gebrekristos, F., Eloul, L., Golden, S., Hemmo, M., Akhtar, A., Schafer, A., & Kohrt, B. A. (in press). Development of a tool to assess competencies of Problem Management Plus facilitators using observed standardised role plays: the EQUIP competency rating scale for PM+. *Intervention*.

Rüdell, K., Bhui, K., & Priebe, S. (2009, March 1, 2009). Concept, development and application of a new mixed method assessment of cultural variations in illness perceptions: Barts explanatory model inventory. *Journal of Health Psychology, 14*(2), 336-347. https://doi.org/10.1177/1359105308100218

Salamanca-Sanabria, A., Richards, D., & Timulak, L. (2019). Adapting an internet-delivered intervention for depression for a Colombian college student population: An illustration of an integrative empirical approach. *Internet Interventions, 15*, 76-86. https://doi.org/10.1016/j.invent.2018.11.005

Sangraula, M., Turner, E. L., Luitel, N. P., van 't Hof, E., Shrestha, P., Ghimire, R., Bryant, R., Marahatta, K., van Ommeren, M., Kohrt, B. A., & Jordans, M. J. D. (2020). Feasibility of Group Problem Management Plus (PM+) to improve mental health and functioning of adults in earthquake-affected communities in Nepal. *Epidemiology and Psychiatric Sciences, 29*, e130-e130. https://doi.org/10.1017/S2045796020000414

Sawaya, H., Atoui, M., Hamadeh, A., Zeinoun, P., & Nahas, Z. (2016, May 30). Adaptation and initial validation of the Patient Health Questionnaire - 9 (PHQ-9) and the Generalized Anxiety Disorder - 7 Questionnaire (GAD-7) in an Arabic speaking Lebanese psychiatric outpatient sample. *Psychiatry Research, 239*, 245-252. https://doi.org/10.1016/j.psychres.2016.03.030

Spitzer, R. L., Kroenke, K., Williams, J. B., & Lowe, B. (2006, May 22). A brief measure for assessing generalized anxiety disorder: the GAD-7. *Archives of Internal Medicine, 166*(10), 1092-1097. https://doi.org/10.1001/archinte.166.10.1092

Tong, A., Craig, J., & Sainsbury, P. (2007). Consolidated criteria for reporting qualitative research (COREQ): a 32-item checklist for interviews and focus groups. *International Journal for Quality in Health Care, 19*(6), 349-357. https://doi.org/10.1093/intqhc/mzm042

van Ommeren, M., Sharma, B., Thapa, S., Makaju, R., Prasain, D., Bhattarai, R., & de Jong, J. (1999, 1999/09/01). Preparing instruments for transcultural research: use of the translation monitoring form with Nepali-speaking Bhutanese refugees. *Transcultural Psychiatry, 36*(3), 285-301. https://doi.org/10.1177/136346159903600304

Willis, G. B. (2004). *Cognitive interviewing: A tool for improving questionnaire design*. Sage Publications.
