# Supplementary material for: Reporting Cultural Adaptation in Psychological Trials – The RECAPT criteria
Source: Clin Psychol Eur. 2021 Nov 23;3(Spec Issue):e6351. doi: 10.32872/cpe.6351 (PMC9670826; doi:10.32872/cpe.6351)
Supplement: Supplement 2 [file cpe-03-6351-s03.docx]

**Reporting Cultural Adaptation in Psychological Trials - The RECAPT criteria**

Eva Heim, Ricarda Mewes, et al., 2021

Manuscript published in Clinical Psychology in Europe, <doi:https://doi.org/10.32872/cpe.6351>

**Template for documenting cultural adaptations of psychological interventions**

**Low quality of reporting:** 4 or fewer

**Moderate quality of reporting:** 5-8 [Minimum criteria]

**High quality of reporting:** 9 or more [Most criteria]

# Set-up

| **Criterion 1: Definition of the target population** | |
| --- | --- |
| **Category** | **Results** |
| Target group |  |

| **Criterion 2: Team and roles**  This table presents relevant information related to the researchers’ backgrounds. Columns can be re-named if necessary. | | | | | |
| --- | --- | --- | --- | --- | --- |
| **Team member** | **Gender** | **Disciplinary background** | **Level / experience /other relevant information** | **Cultural characteristics** | **Role** |
|  |  |  |  |  |  |
|  |  |  |  |  |  |
|  |  |  |  |  |  |
|  |  |  |  |  |  |

# Formative research

| **Criterion 5: Formative research methods** | |
| --- | --- |
| **Category** | **Results** |
| Literature review |  |
| Qualitative methods |  |
| Quantitative methods |  |

| **Criterion 6: Target symptoms, syndromes, needs, and context** | | |
| --- | --- | --- |
| **Category** | **Results** | **Source** |
| Idioms of distress, specific target symptoms |  |  |
| Explanatory and ethnopsychological models |  |  |
| Cultural concepts of distress |  |  |
| Beliefs about the course of the disorder and help-seeking behaviour |  |  |
| Mental health related stigma |  |  |
| Specific needs and other relevant contextual information |  |  |

# Intervention adaptation

| **Criterion 7: Specific treatment elements**  **Criterion 8: Unspecific elements and therapeutic techniques** | | | | | | | | | | | | |
| --- | --- | --- | --- | --- | --- | --- | --- | --- | --- | --- | --- | --- |
| **Decision-Nr.** | **Mechanisms of action including treatment elements, techniques, delivery, surface** | **Original intervention** | **Cultural Processes related to mechanism of action** | **Cultural / contextual adaptation** | **Evidence base**  e.g., literature review, focus groups, qualitative interview | **Quality of evidence**  Strong  Moderate  Weak | **Suggestions from research team** | | | | **State of decision** | |
|  |  |  |  |  |  |  | **Researcher 1** | **Researcher 2** | **Researcher 3** | **Researcher 4** | **pending** | **made** |
|  |  |  |  |  |  |  |  |  |  |  |  |  |
|  |  |  |  |  |  |  |  |  |  |  |  |  |
|  |  |  |  |  |  |  |  |  |  |  |  |  |

| **Criterion 9: Surface adaptations** | | | | | | | | | | | |
| --- | --- | --- | --- | --- | --- | --- | --- | --- | --- | --- | --- |
| **Decision-Nr.** | **Treatment elements, techniques, delivery, surface** | **Original intervention** | **Cultural / contextual adaptation** | **Evidence base**  e.g., literature review, focus groups, qualitative interview | **Quality of evidence**  Strong  Moderate  Weak | **Suggestions from research team** | | | | **State of decision** | |
|  |  |  |  |  |  | **Researcher 1** | **Researcher 2** | **Researcher 3** | **Researcher 4** | **pending** | **made** |
|  |  |  |  |  |  |  |  |  |  |  |  |
|  |  |  |  |  |  |  |  |  |  |  |  |

# Measuring outcomes

| **Criterion 10: Questionnaires and clinical interviews**  **Criterion 11: Implementation measures** | | | |
| --- | --- | --- | --- |
| **Category** | **Instruments used for outcome assessments** | **Translation / validation / adaptation** | **Sources** |
| Clinical Interviews |  |  |  |
| Questionnaires |  |  |  |
| Implementation Measures |  |  |  |
